# Evidence implicating sequential commitment of the founder lineages in the human blastocyst by order of hypoblast gene activation

**DOI:** 10.1242/dev.201522

**Published:** 2023-05-24

**Authors:** Elena Corujo-Simon, Arthur H. Radley, Jennifer Nichols

**Affiliations:** ^1^ Wellcome Trust – MRC Stem Cell Institute, University of Cambridge, Jeffrey Cheah Biomedical Centre, Puddicombe Way, Cambridge CB2 0AW, UK; ^2^ Department of Physiology, Development and Neuroscience, University of Cambridge, Tennis Court Road, Cambridge CB2 3EG, UK; ^3^ Centre for Trophoblast Research, University of Cambridge, Cambridge CB2 3EG, UK

**Keywords:** Hypoblast, Human blastocyst, Inner cell mass, PDGFRA, Lineage acquisition, Entropy sorting

## Abstract

Successful human pregnancy depends upon rapid establishment of three founder lineages: the trophectoderm, epiblast and hypoblast, which together form the blastocyst. Each plays an essential role in preparing the embryo for implantation and subsequent development. Several models have been proposed to define the lineage segregation. One suggests that all lineages specify simultaneously; another favours the differentiation of the trophectoderm before separation of the epiblast and hypoblast, either via differentiation of the hypoblast from the established epiblast, or production of both tissues from the inner cell mass precursor. To begin to resolve this discrepancy and thereby understand the sequential process for production of viable human embryos, we investigated the expression order of genes associated with emergence of hypoblast. Based upon published data and immunofluorescence analysis for candidate genes, we present a basic blueprint for human hypoblast differentiation, lending support to the proposed model of sequential segregation of the founder lineages of the human blastocyst. The first characterised marker, specific initially to the early inner cell mass, and subsequently identifying presumptive hypoblast, is PDGFRA, followed by SOX17, FOXA2 and GATA4 in sequence as the hypoblast becomes committed.

## Teaser

**Summary:** Optimal segregation of human blastocyst founder lineages is essential to establish healthy human pregnancies. Mapping the activation of hypoblast marker genes over time helps to understand how the yolk sac is regulated.

## INTRODUCTION

At implantation, around 8 days after fertilisation (D8), the human blastocyst comprises three lineages: the trophectoderm (TE), epiblast and hypoblast. The TE mediates the uterine connection and forms the placenta. The epiblast is the precursor of the embryo proper. The hypoblast gives rise to the yolk sac (Hertig et al., 1956) and is required to pattern the epiblast to establish the foetus (Mackinlay et al., 2021; Mole et al., 2021). Segregation of these lineages has been well described for the mouse embryo, but emerging data from human embryos implies some divergence of developmental processes between the species. Successful pregnancies following assisted conception are disappointingly low: around 25% of transferred embryos [metrics from the Human Fertilisation and Embryology Authority (HFEA); https://www.hfea.gov.uk/about-us/publications/research-and-data/fertility-treatment-2019-trends-and-figures/]. Understanding how lineages segregate in correct proportions for blastocyst maturation is needed to optimise culture regimes and thereby improve birth rates. Several models have been proposed to explain acquisition of preimplantation lineages in human embryos. The one-step version, based on single-cell RNA-sequencing (scRNA-seq) suggests that TE, epiblast and hypoblast fates are acquired synchronously at around D5 (Petropoulos et al., 2016). The two-step model proposes that, as for the mouse embryo, TE and inner cell mass (ICM) lineages begin segregation at the morula stage, with the hypoblast and epiblast emerging subsequently from the ICM following cavitation (Blakeley et al., 2015; Niakan and Eggan, 2013; Roode et al., 2012). Further scRNA-seq analyses and immunofluorescence (IF) for pan markers of the TE versus ICM support position initiation of the TE programme in morulae via polarity acquisition, activation of phospholipase C, Hippo signalling and GATA3, and nuclear localisation of YAP (Gerri et al., 2020; Meistermann et al., 2021; Stirparo et al., 2018; Zhu et al., 2021) prior to appearance of the epiblast and hypoblast. One report suggested origination of the hypoblast from the epiblast (Meistermann et al., 2021). Using an entropy-based approach for feature selection for published scRNA-seq, we identified an early ICM population from which both the epiblast and hypoblast arise (Radley et al., 2022), as implicated previously (Stirparo et al., 2018).

Specification of the epiblast and hypoblast in human embryos has not been described in detail. Hypoblast induction does not appear to result simply from FGF/ERK signalling from the epiblast, as in mouse (Kuijk et al., 2012; Nichols et al., 2009; Roode et al., 2012). Divergence in the order of gene expression during this segregation in humans compared with that in mice was first investigated using IF (Niakan and Eggan, 2013) and subsequently complemented with scRNA-seq, enabling the identification of 164 genes that are differentially expressed between the two lineages (Blakeley et al., 2015; Yan et al., 2013). A larger dataset identified *LINC00261* as the most highly expressed hypoblast gene; *PDGFRA*, *FGFR2*, *LAMA4*, *HNF1B*, *COL4A1*, *GATA4*, *FN1*, *FRZB*, *AMOTL1* and *DPPA4* were also highlighted (Petropoulos et al., 2016). Hypoblast markers were further separated as ‘early’ for those expressed before the hypoblast is fully specified (*GATA6*, *LRP2* and *ANXA3*) and ‘late’ for genes appearing in the mature lineage (*APOA1*, *COL4A1*, *GDF6*, *RSPO3* and *FST*). OTX2 was discovered to mark the human hypoblast, providing another example of divergence in lineage identity from that of mouse (Boroviak et al., 2018).

We sought to specify the order of appearance of hypoblast-associated gene products, thereby further delineating blastocyst staging and improving embryo quality control. We compared the initiation and downregulation of candidate hypoblast genes and proteins during blastocyst expansion alongside epiblast markers to resolve the sequence of cell fate specification in human embryos and determine whether hypoblast cells arise via conversion of epiblast-specified cells, or from the pluripotent ICM following its segregation from TE.

## RESULTS AND DISCUSSION

### The hypoblast lineage is acquired progressively, concurrent with intensification of SOX17 in ICM cells during blastocyst development

**Fig. 1.**
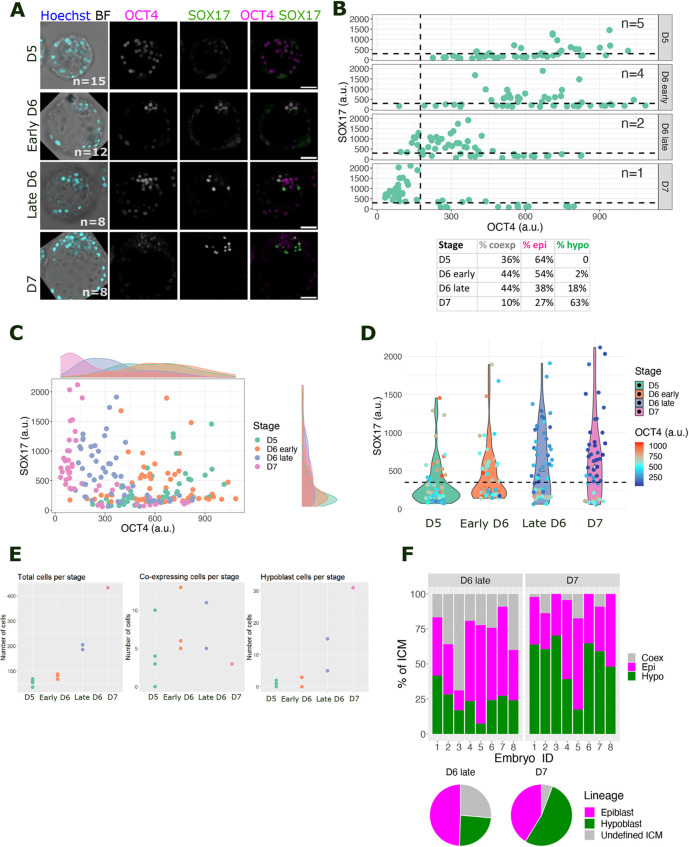
**Tracing the appearance of hypoblast in human blastocysts from D5 to D7.** (A) Representative confocal images of human blastocysts at day (D) 5, early D6, late D6 and D7, immunostained for OCT4 (epiblast, magenta) and SOX17 (hypoblast, green) from *z-*stack combinations of nine consecutive 1 µm single images. *n* indicates the number of embryos analysed per stage. Scale bars: 50 µm. (B) Scatter plots quantifying nuclear intensity of OCT4 (*x*-axis) and SOX17 (*y*-axis) in the ICM. Each dot corresponds to one cell. Dashed lines represent the intensity level/expression threshold calculated at D7, after the TE, epiblast and hypoblast had segregated. a.u., arbitrary units. Table below indicates the percentage of cells belonging to each lineage at the different stages. (C) Scatter plot combining nuclear intensity data from ICM cells at different stages. Marginal density plots showing distribution of data into discrete populations or heterogeneous levels. (D) Violin plot comparing nuclear fluorescence intensity of SOX17 across stages and its co-expression with nuclear OCT4. The dashed line represents the expression threshold for SOX17 calculated at D7. (E) Swarm plot for the absolute number of total cells, OCT4 and SOX17 co-expressing cells, and hypoblast cells per stage. (F) Top panel: stacked bar plot quantifying the percentage of each lineage in individual ICMs at late D6 and D7. Lower panel: pie charts showing the average percentage of each lineage in each stage.

D5 human blastocysts were thawed and fixed immediately after recovery, or following culture to early D6, late D6 or D7 stages. Although still present in the TE at early stages, OCT4 staining was used to track the ICM/epiblast lineage, whereas SOX17 staining identified the hypoblast (Fig. 1A) (Niakan and Eggan, 2013). Embryos were staged using a combination of number of days of culture post fertilisation and morphological landmarks, including zona pellucida thickness, blastocoel size and cell number (Fig. S1). At D5, the embryo ICM comprised 64% OCT4^+^ only cells and 36% co-expressing SOX17 with high levels of OCT4 (Fig. 1A-D; Fig. S2). At early D6, 46% of ICM cells co-expressed OCT4 and SOX17; the detection of 54% cells displaying high OCT4 levels without SOX17 suggests the emergence of epiblast precursors (Fig. 1A-D). At late D6, the ICM could be separated into three populations of cells: epiblast precursors expressing only OCT4 (38%); undefined ICM cells displaying high levels of SOX17 and OCT4 (44%), and hypoblast precursors expressing SOX17 but no OCT4 (18%) (Fig. 1A-E; Fig. S2). At D7, the undefined population was reduced to 10% as it resolved into 27% epiblast and 63% hypoblast cells, distinguishable by the expression of either OCT4 or SOX17. Coincidentally, by this stage, the hypoblast abutted the blastocoel (Fig. 1A). This sequence suggests that nascent human ICMs (D5) possess OCT4^+^ SOX17^−^ cells, whereas by early D6, they co-express markers for the epiblast and hypoblast as SOX17 levels intensify in the double-positive cells, indicating dual progenitor cells within the ICM population poised to form either the epiblast or hypoblast. The same trend was observed in three additional datasets, namely, low or no SOX17 expression in the ICM, then OCT4 and SOX17 co-expression at early and late D6, and, finally, segregation of ICM cells into exclusively OCT4^+^ (epiblast) or SOX17^+^ (hypoblast) lineages (Fig. S2).

The proportion of epiblast and hypoblast (also called primitive endoderm) cells in late mouse blastocysts is largely consistent and maintained at 40%:60% (Saiz et al., 2020, 2016). However, in D7 human embryos, this proportion appeared to vary from 20 to 60% for both the hypoblast and epiblast (Fig. 1F). The percentage of putative epiblast cells in the ICM remained nearly constant from late D6 to D7 (49-41%), suggesting that the OCT4 and SOX17 co-expressing cells at late D6 would have become the hypoblast at D7 (Fig. 1F). The small decrease in the percentage of epiblast cells implies they are fully specified and do not convert to the hypoblast from late D6 to D7.

The progressive increase in hypoblast cells during blastocyst expansion and scarcity of OCT4 and SOX17 co-expressing cells at D5 supports the hypothesis that the hypoblast originates from epiblast cells in human embryos (Meistermann et al., 2021): exclusively OCT4^+^ epiblast cells at D5 subsequently upregulate SOX17, generating the OCT4 and SOX17 co-expressing population at D6 that differentiates into the hypoblast at late D6 and D7. However, our data imply that hypoblast cells could emerge from the subpopulation of OCT4^+^ SOX17^+^ cells rather than by conversion from the epiblast. It is possible that D5 OCT4^+^ cells express an alternative, earlier hypoblast marker than SOX17, classifying them as early ICM cells (Radley et al., 2022). To test this, we investigated hypoblast markers expressed immediately after cavitation.

### Pseudotime analysis of blastocyst development unveils sequential activation of genes specifying the hypoblast lineage

**Fig. 2.**
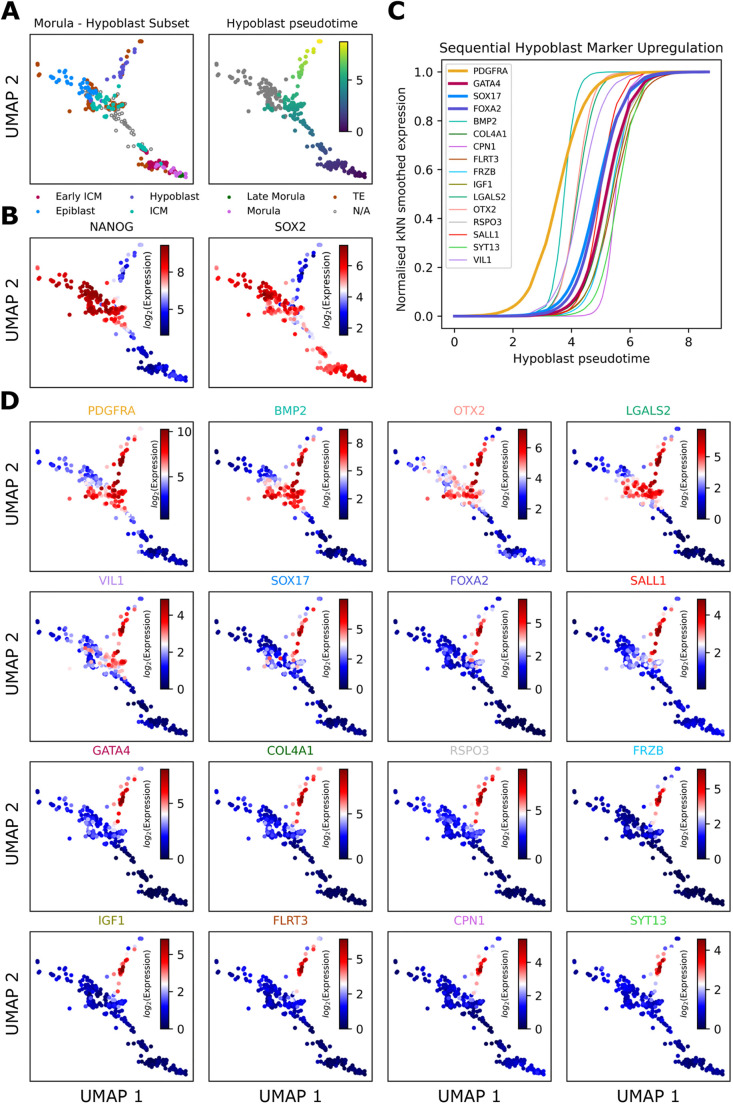
**Pseudotime analysis of published scRNA-seq data from human embryos to establish the order of appearance of hypoblast markers.** (A) Human preimplantation embryo UMAP embedding (Radley et al., 2022) sub-setted down to samples involved during bifurcation to epiblast/hypoblast populations. Left panel: cell type labels defined by Stirparo et al. (2018). Right panel: pseudotime along the hypoblast branch. (B) NANOG and SOX2 distinguish hypoblast and epiblast populations. kNN, k-nearest neighbours. (C) Fitting a logistic curve to the smoothed gene expression against hypoblast pseudotime delineates hypoblast gene activation. (D) Overlays of smoothed gene expression onto UMAP confirms ordered upregulation specific to the hypoblast.

In mouse blastocysts, hypoblast fate is acquired sequentially, originating from an ICM population co-expressing NANOG (epiblast) and GATA6 (hypoblast), which segregate into a random and mutually exclusive arrangement in mid blastocysts (Plusa et al., 2008). Sequential activation of hypoblast markers was investigated using IF, revealing progressive expression of hypoblast transcription factors in the order: GATA6→SOX17→GATA4→SOX7 (Artus et al., 2011). This was confirmed by pseudotime analysis of scRNA-seq from early mouse embryos (Nowotschin et al., 2019). Hypoblast genes in human embryos were classified as either early or late using scRNA-seq (Boroviak et al., 2018). Denoising and feature selection software [functional feature amplification via entropy sorting (FFAVES) and entropy sort feature weighting (ESFW)] expands the potential to detect small temporal differences in the expression of individual genes (Radley et al., 2022). This method was used to characterise the order of appearance of human hypoblast marker genes. We identified specific genes uniquely expressed in hypoblast and absent from the TE and epiblast compartments, beginning by selecting those known to mark the mouse hypoblast (Artus et al., 2011; Chazaud et al., 2006; Plusa et al., 2008). To search for genes not yet associated with the hypoblast, we leveraged a high-resolution scRNA-seq human preimplantation embryo uniform manifold approximation and projection (UMAP) embedding (Radley et al., 2022) generated from published scRNA-seq datasets (Fig. 2A-D; Fig. S3A,B) (Meistermann et al., 2021). Genes were ranked based on the levels of enrichment in the hypoblast according to the UMAP (Fig. 2D; Fig. S3C-F). The order of appearance was obtained by fitting a logistic curve to smoothed gene expression values of the selected hypoblast genes along the hypoblast pseudotime and identifying the mid-point of each logistic curve (Fig. 2C; Fig. S4). Based on this, we suggest the following order:

*PDGFRA→BMP2→OTX2→LGALS2→VIL1→SOX17→FOXA2→SALL1→GATA4→COL4A1→RSPO3→FRZB→IGF1→FLRT3→CPN1→SYT13* (Fig. 2C,D)*.*

The first divergence in the order of gene expression in the human embryo compared with that in the mouse embryo appeared with *GATA6* and *SOX7. GATA6* appears to be a generic human preimplantation extra-embryonic marker expressed in both the TE and hypoblast (Fig. S3D) (Boroviak et al., 2018; Roode et al., 2012). Surprisingly, *SOX7* is absent from human blastocysts, whereas *OTX2* marks the hypoblast in human (Fig. S3C) but not mouse (Boroviak et al., 2018). *FOXA2* appears in the human hypoblast but is absent in mouse mid blastocysts. In late human blastocysts, expression of the FOXA2 protein overlaps with that of SOX17, whereas FOXA2 appears only in a subset of hypoblast cells at this stage in mouse (Blakeley et al., 2015). Thus, according to published data, *PDGFRA*, *BMP2*, *OTX2* and *LGALS2* can be classified as ICM genes in humans; *VIL1*, *SOX17*, *FOXA2* and *SALL1* as marking the mid-stage hypoblast; and *GATA4* marking the mature hypoblast (Fig. 2; Figs S3 and S4). To verify the order of appearance of the selected marker proteins, we performed IF.

### PDGFRA is the first hypoblast marker to appear in the ICM of human blastocysts

**Fig. 3.**
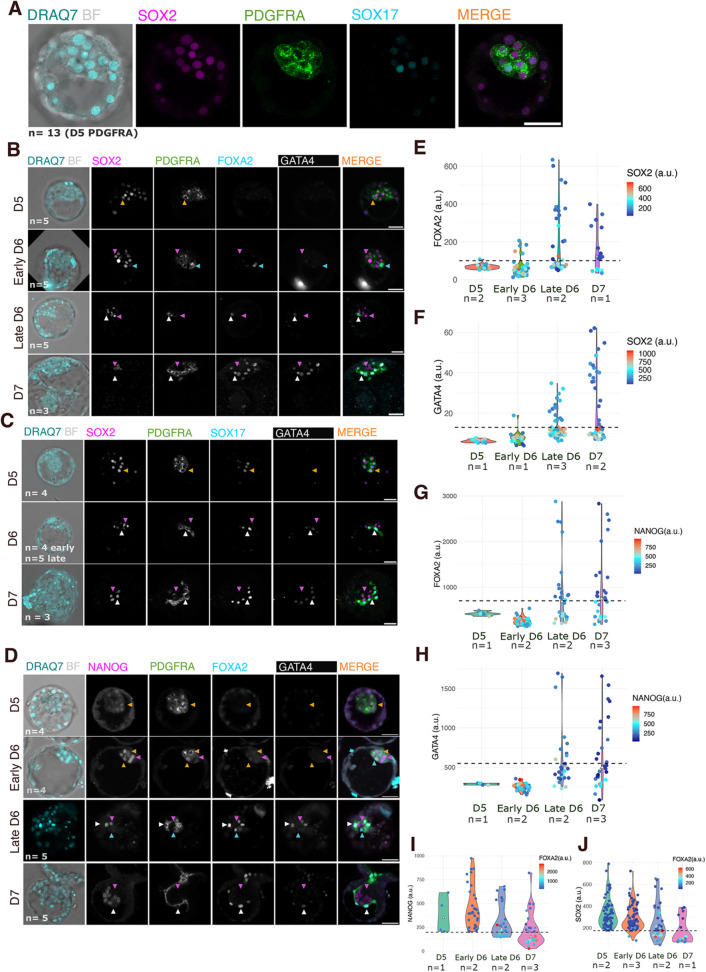
**Validation of hypoblast marker order by immunostaining.** (A) Representative confocal images of a D5 human embryo showing PDGFRA (green) in all ICM cells, with a subset labelled by SOX2 (magenta). DRAQ7 marks nuclei (turquoise). Brightfield (BF) imaging is shown in grey. Scale bar: 50 µm. (B-D) Representative confocal images of D5 to D7 human embryos immunostained for SOX2 (B,C) or NANOG (D) in magenta and PDGFRA (B-D) in green, GATA4 (B-D) in white, SOX17 (C) or FOXA2 (B,D) in cyan. Orange arrowheads indicate SOX2 and/or NANOG co-expressing cells; magenta arrowheads indicate epiblast precursors and epiblast cells; cyan arrowheads indicate hypoblast precursors without GATA4 (FOXA2^+^ GATA4^−^); white arrowheads indicate hypoblast cells. Scale bars: 50 µm. (E,G) Violin plot quantifying FOXA2 across stages and its co-expression with SOX2 (E) or NANOG (G) in the colour map. The dashed line represents the threshold calculated at D7. (F,H) Violin plot quantifying nuclear expression of GATA4 across stages and its co-expression with SOX2 (F) or NANOG (H) in the colour map. The dashed line represents the threshold calculated at D7. (I,J) Violin plot quantifying nuclear expression of NANOG (I) and SOX2 (J) and its co-expression with FOXA2 in the colour map throughout blastocyst development.

Validated antibodies for many of the previously unreported hypoblast markers identified through high-resolution UMAP are not available. Therefore, for the hypoblast, we focused on PDGFRA, SOX17, FOXA2 and GATA4. For the epiblast, we chose to focus on SOX2 and NANOG, as both are lost from hypoblast precursors at D6, in contrast to OCT4, the expression of which persists in the presumptive hypoblast until early D7 (Blakeley et al., 2015; Chen et al., 2009; Niakan and Eggan, 2013; Roode et al., 2012) (Figs 1 and 2B; Fig. S3E). PDGFRA appeared in the membrane of all cells of the early D5 ICM and co-expressed with both SOX2 and NANOG (Fig. 3A-D). At early D6, emerging epiblast precursors downregulated PDGFRA and its expression was completely lost in the epiblast at late D6 while remaining high in the hypoblast (Fig. 3A-D). This is the first indication of a common precursor in the human ICM giving rise to epiblast and hypoblast lineages, whereas distribution of SOX17 was heterogeneous at D5, then widespread in OCT4 and PDGFRA co-expressing ICM cells at early D6 (Fig. 1). SOX17 became specific to the hypoblast at late D6 and was still present at D7 (Fig. 3C). Neither FOXA2 nor GATA4 was expressed at D5 (Fig. 3). FOXA2 expression was detectable earlier than that of GATA4 at D6, based on scRNA-seq and image quantification (Fig. 3B,D-H). GATA4^+^ cells exclusively face the cavity (Guo et al., 2021; Meistermann et al., 2021; Roode et al., 2012), suggesting that its expression commences after physical epiblast:hypoblast, sorting, whereas FOXA2^+^ cells were also observed within the ICM core (Fig. 3B,D). ICM cells can be positive for both NANOG and SOX2 as well as PDGFRA and SOX17 at early D6; however, in later embryos, cells exhibiting high levels of FOXA2 and GATA4 had low levels of SOX2 and very low levels of NANOG (Fig. 3B-J). At late D6, some ICM cells downregulated NANOG/SOX2 and began to upregulate FOXA2 and GATA4, whereas the remaining ICM cells maintained high levels of both epiblast markers (Fig. 3I,J). Once FOXA2 and GATA4 expression was established, SOX2 and NANOG expression was undetectable in these putative determined hypoblast cells. We suggest that the emergence of hypoblast precursors coincides with the onset of FOXA2 expression and downregulation of epiblast markers (Fig. 3).

The presence of PDGFRA in the membrane of all ICM cells at early D5 (Fig. 3A-D) suggests that both epiblast and hypoblast arise from a common pool of ICM cells (Radley et al., 2022), rather than derivation of hypoblast cells from the epiblast (Meistermann et al., 2021). Inclusion of SOX2 and NANOG in our characterization allowed us to determine which population was specified first from the ICM. OCT4^+^ SOX17^−^ cells appeared before OCT4^−^ SOX17^+^ cells at D6 (Fig. 1A), implying that the epiblast is specified before the hypoblast, as suggested (Boroviak et al., 2018). Both SOX2^+^ PDGFRA^−^ and NANOG^+^ PDGFRA^−^ cells were present at D6, confirming earlier appearance of epiblast precursors than the hypoblast counterpart (Fig. 3B-D).

**Fig. 4.**
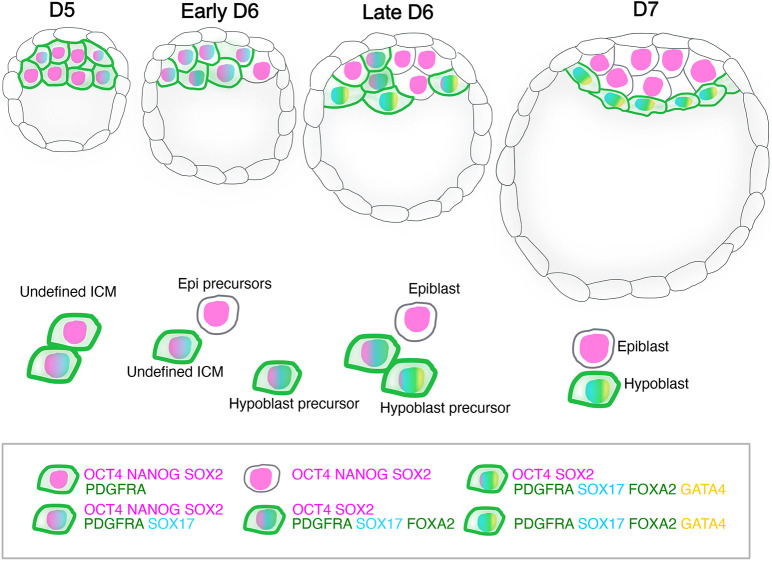
**Schematic of the appearance of epiblast and hypoblast lineages in human blastocysts based on sequential *de novo* expression of hypoblast markers.** At D5, the ICM comprises cells co-expressing OCT4, NANOG, SOX2 and PDGFRA (membrane). At early D6, SOX17 appears in the undefined ICM with PDGFRA, OCT4, SOX2 and NANOG. In parallel, some cells lose PDGFRA and become epiblast (epi) precursors. At late D6, more epiblast cells appear in the ICM and hypoblast precursors emerge, expressing SOX17, FOXA2 and PDGFRA but not NANOG or SOX2. More advanced hypoblast cells beside the blastocoel express GATA4, the last marker to appear. At D7, both lineages are specified and sorted: hypoblast cells beside the blastocoel and the epiblast between the hypoblast and polar TE. Epiblast cells express NANOG, OCT4 and SOX2, whereas PDGFRA, SOX17, FOXA2 and GATA4 mark the hypoblast.

To summarise, our data support the model wherein the epiblast and hypoblast arise from a common progenitor, the ICM (Radley et al., 2022). Although this conclusion was primarily based on the existence of ICM markers, such as LAMA4, that are downregulated in the differentiating ICM, we also investigated hypoblast marker expression at early stages to determine whether an epiblast and hypoblast co-expressing ICM population exists that subsequently resolves into separate lineages. The presence of PDGFRA in all ICM cells at D5 together with OCT4, SOX2 and NANOG indicates that both lineages originate in a population of cells co-expressing epiblast and hypoblast markers (Figs 3 and 4). Our data also strengthen the hypothesis that the epiblast is specified before the hypoblast as NANOG^+^-only or SOX2^+^-only cells appear earlier than hypoblast-only cells (Figs 1, 3 and 4), consistent with conclusions drawn from cell transfer experiments in preimplantation mouse embryos (Grabarek et al., 2012). Early epiblast specification in human blastocysts could result from epiblast factors already expressed in ICM, whereas the hypoblast population is strengthened with *de novo* expression of successive markers from D5 to D7. At D5, only PDGFRA and SOX17 are expressed, contrasting with the high levels of PDGFRA, SOX17, FOXA2 and GATA4 at D7 (Figs 1 and 3). Establishing the order of appearance of hypoblast markers in the context of various levels of epiblast markers during human blastocyst maturation provides an important benchmark for assessing the utility of blastoids constructed from stem cell lines as a model to study the early stages of human embryo implantation *in vitro*, as currently these structures tend to exhibit low hypoblast contribution (Kagawa et al., 2022; Yanagida et al., 2021).

## MATERIALS AND METHODS

### Human embryos

Supernumerary frozen human embryos were donated with informed consent by couples undergoing *in vitro* fertility treatment. Use of human embryos in this research is approved by the Multi-Centre Research Ethics Committee, approval O4/MRE03/44, Integrated Research Application System (IRAS), and licensed by the Human Embryology and Fertilization Authority of the United Kingdom, research license R0178.

Supernumerary frozen blastocysts (D5 and D6) were thawed and cultured in N2B27 medium (Table S1) under mineral oil in a humidified incubator at 37°C, 7% CO_2_ and 5% O_2_ until they reached the desired stage of development from embryonic (E) day 5 to E7. Embryonic stage was assessed based on thinning of the zona pellucida and blastocoele expansion (Fig. S1).

### Immunostaining of human embryos

The zona pellucida of D5 and D6 blastocysts was removed using acid Tyrode's solution (Gibco), before fixation with 4% paraformaldehyde in PBS for 15 min at room temperature. Embryos were rinsed in PBS containing 3 mg/ml polyvinylpyrrolidone (Sigma-Aldrich) (PBS/PVP), permeabilised using 0.25% Triton X-100 (Sigma-Aldrich) in PBS/PVP for 30 min and blocked in blocking buffer comprising PBS supplemented with 0.1% bovine serum albumin, 0.01% Tween 20 (Sigma-Aldrich) and 2% donkey serum for 2 h at room temperature. Primary and secondary antibodies were diluted in blocking buffer (see Table S2). Embryos were incubated in primary antibody solution overnight at 4°C and rinsed three times for 15 min in blocking buffer, before incubation in secondary antibody solution for 1-2 h at room temperature in the dark. Embryos were rinsed in blocking buffer and imaged through a poly-D-lysine-coated Mattek dish (P356-0-14) while submerged in blocking buffer.

### Image acquisition and quantification

Embryos were imaged in a Leica Stellaris 8 confocal microscope and confocal images were converted to 8-bit using FIJI. For objectives and numerical aperture, see Table S3. The Hoechst 33342 channel was used for nuclei segmentation in three dimensions using ZeroCostDL4Mic StarDist via Google colaboratory (Schmidt et al., 2018; Weigert et al., 2018). For details on software training for embryo images, see Kraunsoe et al. (2023).

The segmented image outcome was merged to the original image using FIJI. Integrated density, mean fluorescence intensity and volume per nuclei were measured using FIJI. Data analysis and data representation were performed using R. Scatter and violin plots were generated using the function ‘ggplot2’ and histogram plots were created using ‘ggExtra’. No statistical analysis was performed owing to the low number of embryos per stage per batch.

The expression threshold that separates the TE and ICM, and the ICM into epiblast, hypoblast and precursors was calculated based on the nuclear fluorescence intensity by generating histograms from the data at D7. At this late stage, epiblast and hypoblast markers are only expressed in the ICM, not the TE; moreover, the ICMs are solely formed by the epiblast and hypoblast with completely distinct marker expression, meaning that plotting SOX17 and/or OCT4 generated at least two normal distributions (negative and positive). The point between the two normal distributions was considered the threshold and it was used in early stages to separate the ICM from the TE and, subsequently, epiblast and hypoblast precursors from the undefined ICM population. Because OCT4 is expressed in the TE at D5 at the same level as the ICM, TE cells were removed manually based on their location in the embryo.

### scRNA-seq analysis

scRNA-seq analysis in this work was primarily built upon the high-resolution human preimplantation embryo UMAP generated recently (Radley et al., 2022). A detailed workflow for reproducing the plots in this paper can be found at https://github.com/aradley/Hypoblast_Activation_Paper. In brief, we took the UMAP embedding defined by Radley et al. (2022) and focussed on the cells present in the bifurcation from the morula to the epiblast/hypoblast cell stages. Pseudotime along the hypoblast branch was calculated using the Slingshot R package (Street et al., 2018). Gene expression values were smoothed by taking the average gene expression for each cell and its 30 most similar cells according to the 3700 highly structured genes identified by Radley et al. (2022). Logistic regression curves were fit to the smoothed expression profiles versus the hypoblast pseudotime. The hypoblast pseudotime value that corresponds to the half-way point on the *y*-axis of the logistic curve indicates the tipping point between a gene being active versus inactive.

## Supplementary Material

10.1242/develop.201522_sup1 Supplementary information

## References

[DEV201522C1] Artus, J., Piliszek, A. and Hadjantonakis, A. K. (2011). The primitive endoderm lineage of the mouse blastocyst: sequential transcription factor activation and regulation of differentiation by Sox17. *Dev. Biol.* 350, 393-404. 10.1016/j.ydbio.2010.12.007 21146513 PMC3461954

[DEV201522C2] Blakeley, P., Fogarty, N. M., Del Valle, I., Wamaitha, S. E., Hu, T. X., Elder, K., Snell, P., Christie, L., Robson, P. and Niakan, K. K. (2015). Defining the three cell lineages of the human blastocyst by single-cell RNA-seq. *Development* 142, 3613. 10.1242/dev.131235 26487783 PMC4631772

[DEV201522C3] Boroviak, T., Stirparo, G. G., Dietmann, S., Hernando-Herraez, I., Mohammed, H., Reik, W., Smith, A., Sasaki, E., Nichols, J. and Bertone, P. (2018). Single cell transcriptome analysis of human, marmoset and mouse embryos reveals common and divergent features of preimplantation development. *Development* 145, dev167833. 10.1242/dev.167833 30413530 PMC6240320

[DEV201522C4] Chazaud, C., Yamanaka, Y., Pawson, T. and Rossant, J. (2006). Early lineage segregation between epiblast and primitive endoderm in mouse blastocysts through the Grb2-MAPK pathway. *Dev. Cell* 10, 615-624. 10.1016/j.devcel.2006.02.020 16678776

[DEV201522C5] Chen, A. E., Egli, D., Niakan, K., Deng, J., Akutsu, H., Yamaki, M., Cowan, C., Fitz-Gerald, C., Zhang, K., Melton, D. A. et al. (2009). Optimal timing of inner cell mass isolation increases the efficiency of human embryonic stem cell derivation and allows generation of sibling cell lines. *Cell Stem Cell* 4, 103-106. 10.1016/j.stem.2008.12.001 19200798 PMC3335201

[DEV201522C6] Gerri, C., Mccarthy, A., Alanis-Lobato, G., Demtschenko, A., Bruneau, A., Loubersac, S., Fogarty, N. M. E., Hampshire, D., Elder, K., Snell, P. et al. (2020). Initiation of a conserved trophectoderm program in human, cow and mouse embryos. *Nature* 587, 443-447. 10.1038/s41586-020-2759-x 32968278 PMC7116563

[DEV201522C7] Grabarek, J. B., Żyżyńska, K., Saiz, N., Piliszek, A., Frankenberg, S., Nichols, J., Hadjantonakis, A. K. and Plusa, B. (2012). Differential plasticity of epiblast and primitive endoderm precursors within the ICM of the early mouse embryo. *Development* 139, 129-139. 10.1242/dev.067702 22096072 PMC3231774

[DEV201522C8] Guo, G., Stirparo, G. G., Strawbridge, S. E., Spindlow, D., Yang, J., Clarke, J., Dattani, A., Yanagida, A., Li, M. A., Myers, S. et al. (2021). Human naive epiblast cells possess unrestricted lineage potential. *Cell Stem Cell* 28, 1040-1056.e1046. 10.1016/j.stem.2021.02.025 33831366 PMC8189439

[DEV201522C9] Hertig, A. T., Rock, J. and Adams, E. C. (1956). A description of 34 human ova within the first 17 days of development. *Am. J. Anat.* 98, 435-493. 10.1002/aja.1000980306 13362122

[DEV201522C10] Kagawa, H., Javali, A., Khoei, H. H., Sommer, T. M., Sestini, G., Novatchkova, M., Scholte Op Reimer, Y., Castel, G., Bruneau, A., Maenhoudt, N. et al. (2022). Human blastoids model blastocyst development and implantation. *Nature* 601, 600-605. 10.1038/s41586-021-04267-8 34856602 PMC8791832

[DEV201522C11] Kraunsoe, S., Azami, T., Pei, Y., Martello, G., Jones, K., Boroviak, T. and Nichols, J. (2023). Requirement for STAT3 and its target, TFCP2L1, in self-renewal of naïve pluripotent stem cells in vivo and in vitro. *Biol. Open* 12, bio059650. 10.1242/bio.059650 36504370 PMC9884119

[DEV201522C12] Kuijk, E. W., Van Tol, L. T., Van De Velde, H., Wubbolts, R., Welling, M., Geijsen, N. and Roelen, B. A. (2012). The roles of FGF and MAP kinase signaling in the segregation of the epiblast and hypoblast cell lineages in bovine and human embryos. *Development* 139, 871-882. 10.1242/dev.071688 22278923 PMC3274353

[DEV201522C13] Mackinlay, K. M. L., Weatherbee, B. A. T., Souza Rosa, V., Handford, C. E., Hudson, G., Coorens, T., Pereira, L. V., Behjati, S., Vallier, L., Shahbazi, M. N. et al. (2021). An in vitro stem cell model of human epiblast and yolk sac interaction. *eLife* 10, e63930. 10.7554/eLife.63930 34403333 PMC8370770

[DEV201522C14] Meistermann, D., Bruneau, A., Loubersac, S., Reignier, A., Firmin, J., François-Campion, V., Kilens, S., Lelièvre, Y., Lammers, J., Feyeux, M. et al. (2021). Integrated pseudotime analysis of human pre-implantation embryo single-cell transcriptomes reveals the dynamics of lineage specification. *Cell Stem Cell* 28, 1625-1640.e6. 10.1016/j.stem.2021.04.027 34004179

[DEV201522C15] Mole, M. A., Weberling, A., Fässler, R., Campbell, A., Fishel, S. and Zernicka-Goetz, M. (2021). Integrin β1 coordinates survival and morphogenesis of the embryonic lineage upon implantation and pluripotency transition. *Cell Rep.* 34, 108834. 10.1016/j.celrep.2021.108834 33691117 PMC7966855

[DEV201522C16] Mulas, C., Kalkan, T., Von Meyenn, F., Leitch, H. G., Nichols, J. and Smith, A. (2019). Defined conditions for propagation and manipulation of mouse embryonic stem cells. *Development* 146, dev173146. 10.1242/dev.173146 30914406 PMC6451320

[DEV201522C17] Niakan, K. K. and Eggan, K. (2013). Analysis of human embryos from zygote to blastocyst reveals distinct gene expression patterns relative to the mouse. *Dev. Biol.* 375, 54-64. 10.1016/j.ydbio.2012.12.008 23261930

[DEV201522C18] Nichols, J., Silva, J., Roode, M. and Smith, A. (2009). Suppression of Erk signalling promotes ground state pluripotency in the mouse embryo. *Development* 136, 3215-3222. 10.1242/dev.038893 19710168 PMC2739140

[DEV201522C19] Nowotschin, S., Setty, M., Kuo, Y. Y., Liu, V., Garg, V., Sharma, R., Simon, C. S., Saiz, N., Gardner, R., Boutet, S. C. et al. (2019). The emergent landscape of the mouse gut endoderm at single-cell resolution. *Nature* 569, 361-367. 10.1038/s41586-019-1127-1 30959515 PMC6724221

[DEV201522C20] Petropoulos, S., Edsgard, D., Reinius, B., Deng, Q., Panula, S. P., Codeluppi, S., Plaza Reyes, A., Linnarsson, S., Sandberg, R. and Lanner, F. (2016). Single-cell RNA-seq reveals lineage and X chromosome dynamics in human preimplantation embryos. *Cell* 167, 285. 10.1016/j.cell.2016.08.009 27662094 PMC5628172

[DEV201522C21] Plusa, B., Piliszek, A., Frankenberg, S., Artus, J. and Hadjantonakis, A. K. (2008). Distinct sequential cell behaviours direct primitive endoderm formation in the mouse blastocyst. *Development* 135, 3081-3091. 10.1242/dev.021519 18725515 PMC2768606

[DEV201522C22] Radley, A., Corujo-Simon, E., Nichols, J., Smith, A. and Dunn, S.-J. (2022). Entropy sorting of single-cell RNA sequencing data reveals the inner cell mass in the human pre-implantation embryo. *Stem Cell Rep.* 18, 47-63. 10.1101/2022.04.08.487653 PMC9859930 36240776

[DEV201522C23] Roode, M., Blair, K., Snell, P., Elder, K., Marchant, S., Smith, A. and Nichols, J. (2012). Human hypoblast formation is not dependent on FGF signalling. *Dev. Biol.* 361, 358-363. 10.1016/j.ydbio.2011.10.030 22079695 PMC3368271

[DEV201522C24] Saiz, N., Williams, K. M., Seshan, V. E. and Hadjantonakis, A. K. (2016). Asynchronous fate decisions by single cells collectively ensure consistent lineage composition in the mouse blastocyst. *Nat. Commun.* 7, 13463. 10.1038/ncomms13463 27857135 PMC5120222

[DEV201522C25] Saiz, N., Mora-Bitria, L., Rahman, S., George, H., Herder, J. P., Garcia-Ojalvo, J. and Hadjantonakis, A. K. (2020). Growth-factor-mediated coupling between lineage size and cell fate choice underlies robustness of mammalian development. *eLife* 9, e56079. 10.7554/eLife.56079 32720894 PMC7513828

[DEV201522C26] Schmidt, U., Weigert, M., Broaddus, C. and Myers, G. (2018). *Cell Detection with Star-Convex Polygons*. Cham: Springer International Publishing. 10.1007/978-3-030-00934-2_30

[DEV201522C27] Stirparo, G. G., Boroviak, T., Guo, G., Nichols, J., Smith, A. and Bertone, P. (2018). Integrated analysis of single-cell embryo data yields a unified transcriptome signature for the human pre-implantation epiblast. *Development* 145, dev158501. 10.1242/dev.169672 29361568 PMC5818005

[DEV201522C28] Street, K., Risso, D., Fletcher, R. B., Das, D., Ngai, J., Yosef, N., Purdom, E. and Dudoit, S. (2018). Slingshot: cell lineage and pseudotime inference for single-cell transcriptomics. *BMC Genomics* 19, 477. 10.1186/s12864-018-4772-0 29914354 PMC6007078

[DEV201522C29] Weigert, M., Schmidt, U., Boothe, T., Muller, A., Dibrov, A., Jain, A., Wilhelm, B., Schmidt, D., Broaddus, C., Culley, S. et al. (2018). Content-aware image restoration: pushing the limits of fluorescence microscopy. *Nat. Methods* 15, 1090-1097. 10.1038/s41592-018-0216-7 30478326

[DEV201522C30] Yan, L., Yang, M., Guo, H., Yang, L., Wu, J., Li, R., Liu, P., Lian, Y., Zheng, X., Yan, J. et al. (2013). Single-cell RNA-Seq profiling of human preimplantation embryos and embryonic stem cells. *Nat. Struct. Mol. Biol.* 20, 1131-1139. 10.1038/nsmb.2660 23934149

[DEV201522C31] Yanagida, A., Spindlow, D., Nichols, J., Dattani, A., Smith, A. and Guo, G. (2021). Naive stem cell blastocyst model captures human embryo lineage segregation. *Cell Stem Cell* 28, 1016-1022 e1014. 10.1016/j.stem.2021.04.031 33957081 PMC8189436

[DEV201522C32] Zhu, M., Shahbazi, M., Martin, A., Zhang, C., Sozen, B., Borsos, M., Mandelbaum, R. S., Paulson, R. J., Mole, M. A., Esbert, M. et al. (2021). Human embryo polarization requires PLC signaling to mediate trophectoderm specification. *eLife* 10, e65068. 10.7554/eLife.65068 34569938 PMC8514238

