# Supplementary material for: Evidence implicating sequential commitment of the founder lineages in the human blastocyst by order of hypoblast gene activation
Source: Development. 2023 May 24;150(10):dev201522. doi: 10.1242/dev.201522 (PMC10233721; doi:10.1242/dev.201522)
Supplement: Supplementary information [file develop-150-201522-s1.pdf]

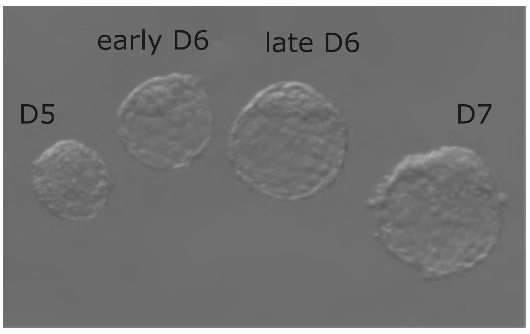

Fig. S1. Human embryo staging from D5 to D7. Representative bright field image of human embryos at D5, early D6, late D6 and D7. Differences in global size and blastocoel size were used to stage the embryos together with zona thickness (removed prior to image capture) and total cell number calculated following image analysis.

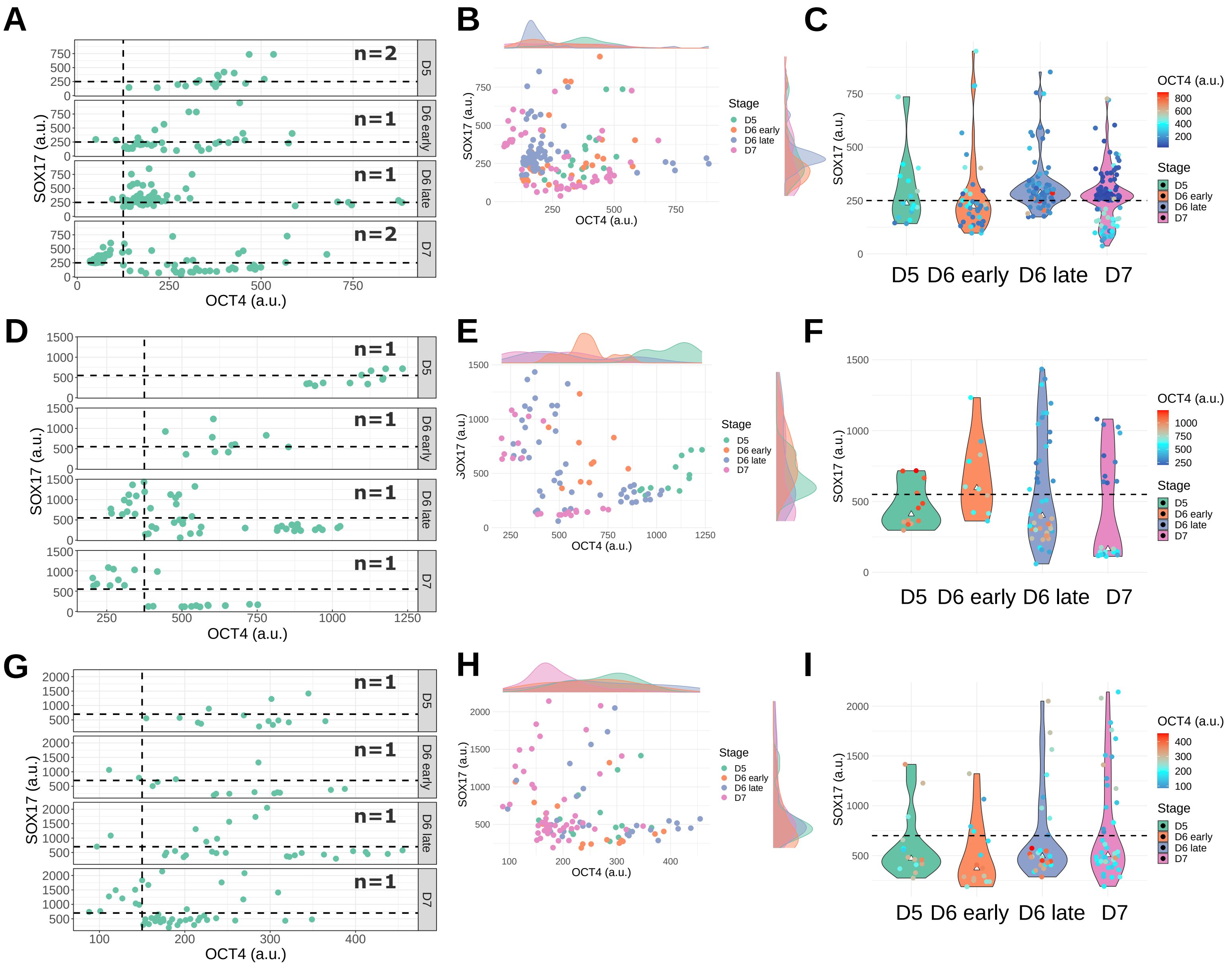

J

Stage	% coexp	% epi	% hypo
D5	47%	53%	0
D6 early	28%	67%	5%
D6 late	77%	19%	4%
D7	10%	29%	61%

corresponding to A-C

Stage	% coexp	% epi	% hypo
D5	33%	67%	0%
D6 early	64%	36%	0%
D6 late	17%	59%	24%
D7	4%	57%	39%

corresponding to D-F

Stage	% coexp	% epi	% hypo
D5	21%	79%	0%
D6 early	13%	73%	0%
D6 late	22%	70%	8%
D7	17%	18%	65%

corresponding to G-H

Fig. S2. Additional imaging batches were examined to confirm the timing of hypoblast appearance in human embryos. Batch 2: **A-C**; Batch 3: **D-F**; Batch 4: **G-I**. **A,D,G**. Scatter plots quantifying the nuclear intensity of OCT4 (x-axis) and SOX17 (y-axis) throughout blastocyst stages in the ICM in individual cells. Dashed lines represent the expression threshold calculated at D7. "n" refers to number of embryos analysed per stage. **B,E,H.** Scatter plots combining the quantification of OCT4 and SOX17 nuclear intensity per cell throughout all of the stages in blastocyst development (D5 to D7). Marginal density plots on the side and on top of the scatter plot show the data distribution that varies between discrete or heterogeneous populations. **C,F,I.** Violin plots showing the changes in SOX17 nuclear intensity across blastocyst stages and its co-expression with OCT4 per cell (colour-map). The dashed line represents the expression threshold for SOX17. **J.** Table showing the percentage of cells belonging to each lineage at the different stages of blastocyst development.

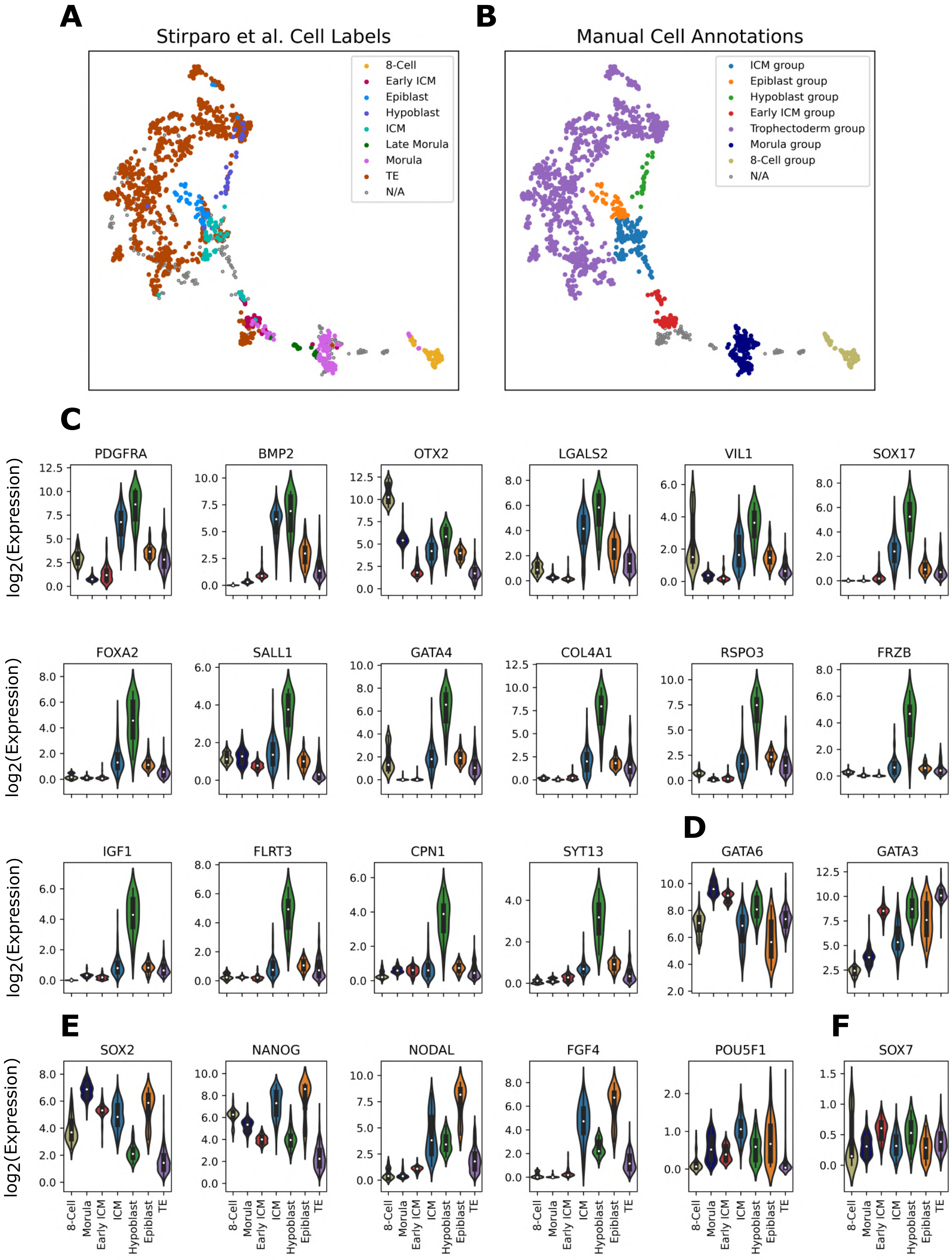
A
Stirparo et al. Cell Labels
8-Cell
Early ICM
Epiblast
Hypoblast
ICM
Late Morula
Morula
TE
N/A
B
Manual Cell Annotations
ICM group
Epiblast group
Hypoblast group
Early ICM group
Trophectoderm group
Morula group
8-Cell group
N/A
C
PDGFRA
BMP2
OTX2
LGALS2
VIL1
SOX17
FOXA2
SALL1
GATA4
COL4A1
RSPO3
FRZB
IGF1
FLRT3
CPN1
SYT13
D
GATA6
GATA3
E
SOX2
NANOG
NODAL
FGF4
POU5F1
F
SOX7
log2(Expression)
8-Cell
Morula
Early ICM
ICM
Hypoblast
Epiblast
TE

Fig. S3. Marker expression across all cells of the human pre-implantation embryo, E3-E7. **A, B**. Human pre-implantation embryo UMAP taken from Radley et al., 2022 where samples are labelled either by the cell type labels provided by Stirparo et al., 2018 (A) or manually labelled (B) based on the UMAP, cell type marker expression and the labels provided by Stirparo et al., 2018. **C-F.** Violin plots for different marker groups of interest, based on the groupings in B**. C**. Hypoblast markers that were presented in Figure 2, ordered from left to right in by their order of activation along the hypoblast pseudotime. **D.** Trophectoderm markers. **E.** Epiblast markers. **F.** Negative control with low expression throughout the human pre-implantation embryo.

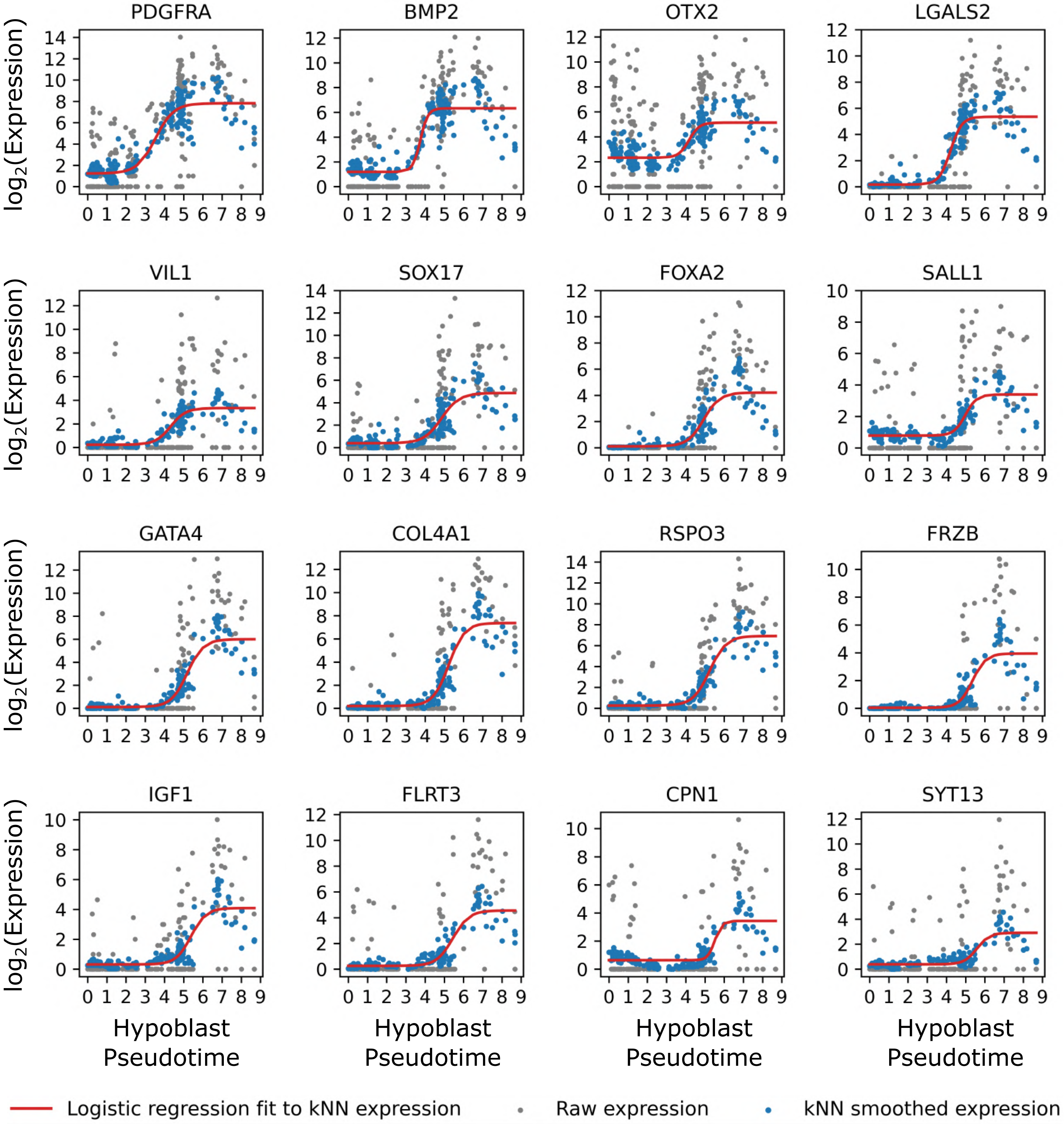

Fig. S4. Logistic regression of gene expression profiles along the hypoblast pseudotime. We use the hypoblast pseudotime generated through the Slingshot software (Figure 2A) to fit a logistic curve to the expression profiles of genes of interest. A useful output of using a logistic curve is that the results provide a clear rationale for separating cells where a gene is considered inactive from cells where a gene is considered active. To fit a logistic curve, we first smooth the raw expression values (grey) of each cell by looking at their neighbourhood of k=30 cells and taking the average expression. We then use these smoothed expression values (blue) to fit a logistic curve, using the scipy.optimise.curve_fit function in Python. The resulting logistic curves are shown in red.

Table S1. N2B27 media recipe

Reagent	Provider – Cat no	Final concentration
DMEM/F-12	Thermo Fisher Scientific – 21331-020	
Neurobasal	Thermo Fisher Scientific - 21103049	
B27 (50×)	Invitrogen - 17504044	0.5×
N2	Made in house (Mulas et al., 2019)	1×
β-mercaptoethanol (50 mM)	Thermo Fisher Scientific - 31350-010	50 µM
L-glutamine (200 mM)	Thermo Fisher Scientific - 25030081	2 mM

Table S2. Antibodies used for immunofluorescence

Antibody	Supplier	Cat no	Dilution
PDGFRα	Abcam	#ab203491 RRID:AB_2892065	1:200
SOX17	R&D System	#AF1924 RRID:AB_355060	1:200
GATA4	EBioscience	#14-9980-82 RRID:AB_763541	1:200
FOXA2/HNF-3β	R&D System	#AF2400 RRID:AB_2294104	1:200
SOX2	Santa Cruz Biotechnology	#sc-365823 RRID:AB_10842165	1:200
OCT4	Santa Cruz Biotechnology	#sc-5279 RRID:AB_628051	1:200
DRAQ 7™	Thermo Fisher Scientific	#D15105	1:1000
Donkey anti-goat 405	Abcam	#ab175664 RRID:AB_2313502	1:500
Alexa Fluor donkey anti-rabbit 488	Thermo Fisher Scientific	#A32790 RRID:AB_2762833	1:500
Alexa Fluor donkey anti-mouse 555	Thermo Fisher Scientific	#A-31570 RRID:AB_2536180	1:500
Alexa Fluor donkey anti-rat 647	Thermo Fisher Scientific	#A78947 RRID:AB_2910635	1:500

Development • Supplementary information

Table S3. Objectives and numerical aperture of embryo imaging in Leica Stellaris microscope

Figure	Objective	Numerical Aperture	Objective Name
1, S2	40× water	1.1	HC PL APO CS2 40×/1.10 WATER
3	40× oil	1.3	HC PL APO CS2 40×/1.30 OIL
