# Supplementary material for: Evidence implicating sequential commitment of the founder lineages in the human blastocyst by order of hypoblast gene activation
Source: Development. 2023 May 24;150(10):dev201522. doi: 10.1242/dev.201522 (PMC10233721; doi:10.1242/dev.201522)
Supplement: Supplementary file 1 [file develop-150-201522_review_history.pdf]

Evidence implicating sequential commitment of the founder lineages in the human blastocyst by order of hypoblast gene activation

Elena Corujo-Simon, Arthur H. Radley and Jennifer Nichols
DOI: 10.1242/dev.201522

Editor: Liz Robertson

Review timeline
Original submission: 10 December 2022
Editorial decision: 18 January 2023
First revision received: 31 March 2023
Accepted: 19 April 2023

Original submission

First decision letter

MS ID#: DEVELOP/2022/201522

MS TITLE: Evidence implicating sequential commitment of the founder lineages in the human blastocyst by order of hypoblast gene activation

AUTHORS: Elena Corujo-Simon, Arthur H Radley and Jennifer Nichols

I have now received all the referees' reports on the above manuscript, and have reached a decision. The referees' comments are appended below, or you can access them online: please go to BenchPress and click on the 'Manuscripts with Decisions' queue in the Author Area.

As you will see, the referees express considerable interest in your work, but have some significant criticisms and recommend a revision of your manuscript before we can consider publication. If you are able to revise the manuscript along the lines suggested, which may involve further experiments, I will be happy receive a revised version of the manuscript. Your revised paper will be re-reviewed by one or more of the original referees, and acceptance of your manuscript will depend on your addressing satisfactorily the reviewers' major concerns. Please also note that Development will normally permit only one round of major revision. If it would be helpful, you are welcome to contact us to discuss your revision in greater detail. Please send us a point-by-point response indicating your plans for addressing the referee’s comments, and we will look over this and provide further guidance.

Please attend to all of the reviewers' comments and ensure that you clearly highlight all changes made in the revised manuscript. Please avoid using 'Tracked changes' in Word files as these are lost in PDF conversion. I should be grateful if you would also provide a point-by-point response detailing how you have dealt with the points raised by the reviewers in the 'Response to Reviewers' box. If you do not agree with any of their criticisms or suggestions please explain clearly why this is so.

© 2023. Published by The Company of Biologists under the terms of the Creative Commons Attribution License (https://creativecommons.org/licenses/by/4.0/).

Reviewer 1

Advance summary and potential significance to field

This is an interesting and well-executed work, in which the authors use previously generated single-cell RNA-seq data from human preimplantation embryos, to identify genetic components and markers involved in the primitive endoderm/hypoblast lineage.

Comments for the author

The main issue with this manuscript is that it is basically a recapitulation of what is already known regarding hypoblast specification in human blastocysts. Even if initial studies (i.e., Petropoulus et al., 2016) proposed a model where simultaneous specification of all three blastocyst lineages (trophectoderm, epiblast and hypoblast) occurs, multiple studies, including those of the authors of this work, have shown that this is a sequential process much as in the mouse.

Many of the finding of this work have already been shown before. For example, the authors nicely detail the dynamics of OCT4 and SOX17 expression in the early human embryo, but this only provides more fine-grained detail to what has already been published (i.e., Blakeley et al., 2015). Similarly, the computational analysis used to identify the sequence of gene activation in the hypoblast (Entropy Sorting) is of interest, but has been already described by the same authors (Radley et al. 2022).

Surely, the most interesting addition to our current understanding of hypoblast specification in humans is the ordered list of gene expression shown in Fig. 2C. However, the authors only address protein expression of four of them (PDGFRA, SOX17, FOXA2 and GATA4) because of lack of adequate reagents for others. Again, the patterns of expression of all four have already been documented before.

Overall, this manuscript fails to provide additional insight into the functions of the newly identified hypoblast-specific genes, or system-wide predictions about the hypoblast-specifying network in humans, which would be requisites for publication in Development of research papers involving genome-wide screens such as this.

Reviewer 2

Advance summary and potential significance to field

This study provides new information on the sequence of genes activated during human hypoblast specification, based on analysis of previously published gene expression data, and newly provided protein colocalisation studies, and identifies PDGFRA as the earliest hypoblast specific marker in humans. Given the scarcity of available data on early lineage specification in human embryos, especially concerning hypoblast, it is a valuable new piece of information and important reference for future studies.

Comments for the author

- Please provide numbers of samples/embryos analysed. This information can be partially found in fig. 1, but missing in most of the cases. Fig. 1A suggests that only 1 D7 embryo was analysed, while fig. 1F has 8 samples.
- Please provide the results of your analysis throughout the manuscript – for example, what is the percentage or number of >mostly OCT4+< cells (l105), >majority of ICM cells< (l108), >some OCT4+SOX17- cells< (l.115) etc.
- Authors use OCT4 as an epi marker, even though this results in erroneous inclusion of TE cells in their epi count (of which they are aware). TE cells should be excluded based on specific TE marker staining. If that`s not possible at this point (given the limitation of human embryo study), OCT4+ cells in TE should be excluded from epi count based on their position in the embryo/TE
- Authors use OCT4, SOX2 and NANOG as markers of epi, noting that their expression patterns differ in human embryos. Please provide more information (based on your study or existing

© 2023. Published by The Company of Biologists under the terms of the Creative Commons Attribution License (https://creativecommons.org/licenses/by/4.0/).

literature) on these differences, and specifically what conclusions can you draw from differential co-expression of studied hypo-markers with those epi markers. OCT4 is used as an epi marker, but it has been shown to be necessary for differentiation of all three lineages in human embryos.
- Conclusions: l. 223 > Analysis of marker expression may be correlated with blastocyst morphology to help identify the best embryos for transfer to patients < . As much as marker expression analysis provides valuable information about mechanisms of embryonic development, currently it can't be used as a tool for assessing embryo quality in clinical applications.
Reference to other data
- L.114 >as in the mouse embryo< - what stage of mouse embryonic development are you referring to, and which publication
- L.55 – yolk sac required to pattern the epi – did you perhaps mean hypo required… ? If so, please provide additional references. In addition to TE and hypo, short info on epi function should be also provided Other suggestions
- The publication would benefit from discussing these findings with available data on hypoblast formation in other mammals, in addition to mouse, to potentially highlight interspecific similarities.
- The organization of presented data seems confusing (to me). Please maybe consider starting your results presentation from gene expression analysis?
- L.261 refers to unpublished methods – if still unpublished, please provide more detailed information in this manuscript

Reviewer 3

Advance summary and potential significance to field

Mechanisms that regulate the segregation of the human hypoblast have not been elucidated, despite their fundamental importance for understanding human development and to inform stem cell derived models of early embryonic or extraembryonic cell types. There is widespread interest in the area, especially with the development of advanced integrated stem cell models of human embryos that are notably deficient in hypoblast specification.

There are suggestions that the epiblast, hypoblast and trophectoderm all arise simultaneously (e.g. Petropolous et al., 2016) at the blastocyst stage. Alternatively, it has been proposed that the hypoblast segregates from either the epiblast (Meistermann et al., 2021) or an unspecified inner cell mass (similar to the mouse). In this manuscript the authors perform immunofluorescence analysis of human embryos to investigate the timing of the initiation and segregation of molecular markers of the epiblast and hypoblast.

The take home messages are: 1) the epiblast and hypoblast arise from common ICM progenitor 2) the molecular hierarchy of hypoblast transcription factor expression is PDGFRA expression, followed by SOX17, FOXA2 and GATA4.

Point 1) is important because there has been some confusion that arose from the Meistermann scRNA-seq analysis (and other scRNA seq papers) where the ICM is poorly defined transcriptionally. However, the authors cover some of this in a recent informative paper in a Stem Cell Reports by re-analysing the Meistermann single cell data: https://www.sciencedirect.com/science/article/pii/S2213671122004568?via%3Dihub. In this manuscript the analysis has been expanded to focus on the hypoblast. We think it's interesting to note that while there is just one panel of staining in the Stem Cell Reports paper, the n number of each stage is considerably higher than in this manuscript.

Point 2) is reminiscent of a publication from Plusa et al 2008: https://doi.org/10.1242/dev.021519 which was also published in Development. This has been an impactful and highly cited study in the field. The biggest difference is that Plusa et al paper contained live-imaging of reporters for Pdgfra and was therefore able to correlate timing, gene expression and morphological formation of the hypoblast layer (but notably, the Plusa et al paper didn't have scRNA-seq analysis, because it hadn't yet been developed).

© 2023. Published by The Company of Biologists under the terms of the Creative Commons Attribution License (https://creativecommons.org/licenses/by/4.0/).

Comments for the author

Major points:

We felt that the approach on this manuscript is sound, but with small n numbers, and human embryos being variable, we were not sure whether conclusions could be drawn with certainty with regards to timing of staining (and epiblast/hypoblast ratios). The authors noted that “No statistical analysis was performed due to the low number of embryos per stage per batch” (line 266) – and we therefore wondered if the authors had managed to obtain more embryos for staining that would allow them to perform statistical analysis. If so, this would strengthen the manuscript and conclusions. Also, the novelty in staining of markers and timing/comparison of OCT4, SOX2, NANOG, GATA4, SOX17, OTX2, FOXA2 have been described before.

We think it would be important for the finding from this manuscript to be out there, and that there is nothing fundamentally wrong with the paper. The only limitation is that it is descriptive. That said it provides protein expression data to address an important and controversial topic in the field and will therefore be a welcome addition to inform our understanding of the mechanisms underlying the second cell fate specification in human development.

Minor points:
1. Please provide details of the objectives and numerical aperture in the methods section.
2. The methods for software training for embryo images could not be evaluated because the publication Kraunsoe et al. is not yet accessible. In the future it would be helpful to have a few lines describing this further.
3. Consistency in labelling is needed (for example Epi, epi, Hypo, hypo are used at different points throughout the manuscript and methods section)
4. Line 230 - 232: fix the text here where the boxes are errors.
5. Detail are needed of how the in house N2B27 is made. This is the basis of the human embryo culture media used.
6. Line 58: cite the HFEA reference metrics used in this sentence.
7. Line 70: Remove the reference Zhu and Zernicka-Goetz, 2020 in this sentence – this is a review on the regulative mechanisms of mammalian development and speaks to Carm1 paraspeckles; similarly we don’t believe the Meistermann does not investigate IF markers that distinguish TE versus ICM, nor do they investigate polarity acquisition or Hippo signalling therefore this reference should also be removed here.
8. Lines 79-82: reference Blakeley et al 2015 (see Fig. 5) is needed where the enrichment of transcripts in the PrE/hpoblast such as GATA4, GATA6, SOX17, PDGFRA, COL4A1, SPARC and FOXA2 were noted.

First revision

<u>Author response to reviewers' comments</u>

Reviewer 1 Advance Summary and Potential Significance to Field:
This is an interesting and well-executed work, in which the authors use previously generated single-cell RNA-seq data from human preimplantation embryos, to identify genetic components and markers involved in the primitive endoderm/hypoblast lineage.

We thank the reviewer for the positive summary of our work.

Reviewer 1 Comments for the Author:
The main issue with this manuscript is that it is basically a recapitulation of what is already known regarding hypoblast specification in human blastocysts. Even if initial studies (i.e., Petropoulus et al., 2016) proposed a model where simultaneous specification of all three blastocyst lineages (trophectoderm, epiblast and hypoblast) occurs, multiple studies, including those of the authors of this work, have shown that this is a sequential process much as in the mouse.

© 2023. Published by The Company of Biologists under the terms of the Creative Commons Attribution License (https://creativecommons.org/licenses/by/4.0/).

We appreciate the reviewer's knowledge of the field, but we are disappointed that they do not recognise the advance in understanding of early developmental processes specific to human embryos that our new short manuscript reports. Specifically, we have uncovered novel aspects of human hypoblast specification and maturation by mapping the appearance of validated hypo markers in relation to expression of epi markers that will ultimately enable definition of the sequence of gene activation necessary to produce a normal expanded blastocyst. Even using the small selection of known hypo markers for which validated antibodies are available we have identified significant divergence in the sequence of events leading to mature hypo compared with the mouse. This is an important step in advancing understanding of how founder lineages need to segregate in the human embryo to allow successful pregnancy. Ultimately, such advances may help influence design of culture regimes to improve live births after assisted conception treatments from the current rate of 25% of embryos transferred.

In a previous well-planned and executed study, using SOX17 as the best characterised marker for human hypo, Meistermann et al., 2021 proposed that this lineage is specified as a derivative of the epi. In our Fig. 1, we recognised how such a conclusion could be reached if SOX17 is regarded as an early hypo marker, as it is in the mouse. By performing immunostaining on a range of stages of human embryos, we found that SOX17 does not appear until late D5, approaching the mid-blastocyst stage, beginning in only a small subset of ICM cells. Our work is the first to demonstrate PDGFRA in the early D5 human blastocyst (see Fig. 3), before SOX17. FOXA2 and GATA4 then appear sequentially after SOX17 (Fig. 1/S2). Here, we sought to build on the findings from Radley et al., 2022 to determine which other known hypo markers are expressed in the early ICM, in order to complement previously published reports characterising appearance of epi markers (eg, Blakely et al., 2015) and thereby determine the temporal relationship between appearance of hypo and epi to characterise the process of specification and commitment in the context of embryo morphology. As well as its potential to be used as a bench mark to improve culture regimes for human IVF-derived embryos, this knowledge will be important for validation of experiments constructing blastoids from pluripotent stem cell lines, which are already gaining popularity as a model system for early human development.

Many of the finding of this work have already been shown before. For example, the authors nicely detail the dynamics of OCT4 and SOX17 expression in the early human embryo, but this only provides more fine-grained detail to what has already been published (i.e., Blakeley et al., 2015). Similarly, the computational analysis used to identify the sequence of gene activation in the hypoblast (Entropy Sorting) is of interest, but has been already described by the same authors (Radley et al. 2022).

We thank the reviewer for recognising our analysis as "detailed". However, we disagree that it adds only fine-grained detail because, to our knowledge, ours is the first report to present single cell image quantification of human embryos and apply it as a readout of signal intensity (Fig. 1). By quantifying protein dynamics between OCT4 and SOX17, we revealed a progression from a gradual increase of SOX17 expression in OCT4+ cells, then mutually exclusive expression of SOX17 and OCT4 in hypo and epi respectively. We also quantified at the single cell level the dynamics of SOX2 and NANOG (epi markers) with FOXA2 and GATA4, which we established as hypo markers appearing after SOX17 and PDGFRA. We show in the scatter plots (Fig. 3) that co-expression of FOXA2 and GATA4 occurs in a small proportion of late ICM cells. We consider the strength of our present manuscript a worthwhile and timely complement to the work presented in Radley et al., 2022, since our immunostaining for markers of both lineages in a series of embryo stages allows visualisation and basic quantification of cells expressing various marker proteins in combination, comparable directly to the stage and morphology of each blastocyst. By manually counting the number of epi, hypo and co-expressing cells in late D6 and D7, we discovered that the epi population does not decrease substantially, while the hypo population is enlarged from the pool of ICM cells co-expressing markers for both lineages. This finding adds weight to the hypothesis that both epi and hypo emerge from a common precursor pool of cells, the "ICM". This analysis was enhanced by use of the entropy sorting software tool developed by Radley, which we are also using for other studies in the lab.

Surely, the most interesting addition to our current understanding of hypoblast specification in humans is the ordered list of gene expression shown in Fig. 2C. However, the authors only address protein expression of four of them (PDGFRA, SOX17, FOXA2 and GATA4) because of lack of

© 2023. Published by The Company of Biologists under the terms of the Creative Commons Attribution License (https://creativecommons.org/licenses/by/4.0/).

adequate reagents for others. Again, the patterns of expression of all four have already been documented before.

We agree that a major addition to our current understanding of hypo specification in humans is establishing the order in which hypo lineage markers appear in the embryo. Because of the scarcity of validated antibodies and human embryos, our aim was to clarify the order of expression of those available in relation to one another during development. Our choice of PDGFRA as a marker for emerging human hypo indeed revealed that specification towards hypo begins earlier than was previously suggested by use of SOX17, FOXA2 or GATA4 staining and reinforced the proposed existence of a common ICM progenitor population for both epi and hypo.

Overall, this manuscript fails to provide additional insight into the functions of the newly identified hypoblast-specific genes, or system-wide predictions about the hypoblast-specifying network in humans, which would be requisites for publication in Development of research papers involving genome-wide screens such as this.

We beg to disagree with the reviewer that our manuscript is intended to be a genome-wide screen. We hope that our response to the criticisms outlined above explain our intention. This short report is largely descriptive, but provides new information contributing to understanding gene function during early lineage specification by evoking a novel temporal aspect.

Reviewer 2 Advance Summary and Potential Significance to Field:
This study provides new information on the sequence of genes activated during human hypoblast specification, based on analysis of previously published gene expression data, and newly provided protein colocalisation studies, and identifies PDGFRA as the earliest hypoblast specific marker in humans. Given the scarcity of available data on early lineage specification in human embryos, especially concerning hypoblast, it is a valuable new piece of information and important reference for future studies.

We thank the reviewer for this positive summary of our findings.

Reviewer 2 Comments for the Author:
-Please provide numbers of samples/embryos analysed. This information can be partially found in fig. 1, but missing in most of the cases. Fig. 1A suggests that only 1 D7 embryo was analysed, while fig. 1F has 8 samples.

We thank the reviewer for noticing this difference, which we agree, we had not properly explained in the manuscript. The apparent discrepancy arises because we present figures referring to the TOTAL number of embryos immunostained for the study. However, the number of ‘normal’ embryos retrieved per thawing session is variable, and since we fix, stain and image them as soon as possible, we anticipate some variability of the subsequent analysis between datasets (eg, laser power, signal intensity). For this reason, we avoided comparing datasets generated on different days directly for the figures. We have clarified this strategy in the revised manuscript and added the total numbers exhibiting the same trend from additional biological repeats and hope the numbers and statistical power for our conclusions are more comprehensive now. To summarise, for Fig. 1, 8 D7 human embryos were stained for OCT4 and SOX17. In Fig. 1F we manually counted the cells per embryo in FIJI as at this stage epi and hypo are already specified and can be easily recognised. For Fig. 1B-E, we show the quantification of one dataset that was stained and imaged in one session, but which resulted in obtaining only one D7 embryo. Additional embryo sets were analysed in Fig. S2. Instead of adding all of the datasets and normalising the data, which would have required modification of the raw values, we opted to repeat the analysis in the smaller datasets as a means to determine whether the order of marker protein expression (appearance, co-expression and segregation) was consistent. To clarify this in the text for Figs. 1, 3 and S2, we now have inserted the total number of embryos immunostained for each combination of antibodies under the BF+Hoechst image. We also included the total number of embryos used per stage in a dataset stained and imaged at the same time in the scatter plot.

-Please provide the results of your analysis throughout the manuscript – for example, what is the percentage or number of >mostly OCT4+< cells (l105), >majority of ICM cells< (l108), >some OCT4+SOX17- cells< (l.115) etc.

© 2023. Published by The Company of Biologists under the terms of the Creative Commons Attribution License (https://creativecommons.org/licenses/by/4.0/).

We thank the reviewer for pointing this out and have added the percentage in the text for Fig. 1. In the case of Fig. S2, we placed the numbers in three tables at the bottom. In so doing, we noticed that the co-expression population does not decrease much in the transit of early to late D6, which we have now revised in the text. We are grateful to the reviewer for prompting us to explore the dynamics further through calculating the percentage values. Early and late D6 appear to be dynamic stages with some temporal variability in acquisition of co-expression. The dataset presented in Fig. 1 was selected based on the large number of early embryos available from that round of thawing.

-Authors use OCT4 as an epi marker, even though this results in erroneous inclusion of TE cells in their epi count (of which they are aware). TE cells should be excluded based on specific TE marker staining. If that`s not possible at this point (given the limitation of human embryo study), OCT4+ cells in TE should be excluded from epi count based on their position in the embryo/TE

As the reviewer points out, adding a TE marker is not possible for the embryos in question since we have already used all available channels. But following this helpful feedback, we have removed the cells that belong to the TE at D5 and early D6 based on their location in the embryo in all our datasets. This has improved visualization in the scatter plots of the ICM at D5 and early D6.
Due to TE and ICM occupying the same section of the scatter plot at these early stages (Figure 1) we have decided to remove the TE and show the ICM only population throughout the manuscript for clarity.

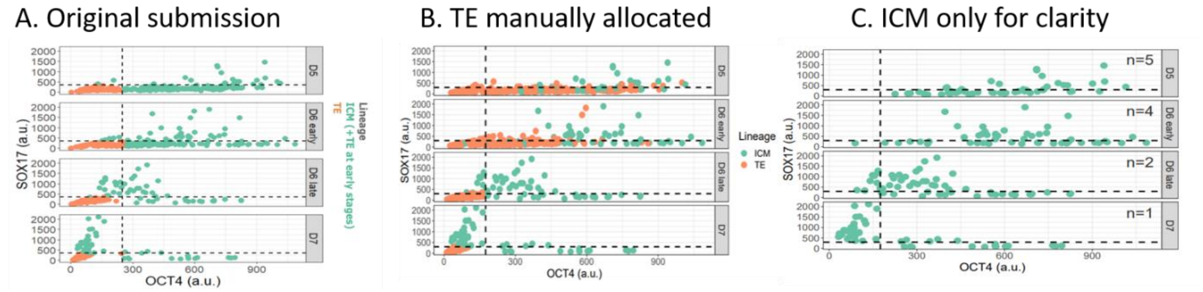

Figure 1. Scatter plots to show lineage populations in the early embryo. A. In the original submission, TE and ICM were separated based on the expression threshold for OCT4 and SOX17 calculated at late D6 and D7. Since OCT4 is also expressed at the same levels in ICM and TE at early D5, the ICM population contained TE cells. B. TE and ICM at early stages are manually labelled based on their position in the embryo rather than their levels of OCT4/SOX17, the threshold is now calculated based solely on D7 data. C. For the final submission, we have removed the TE cells from the scatter plots.

-Authors use OCT4, SOX2 and NANOG as markers of epi, noting that their expression patterns differ in human embryos. Please provide more information (based on your study or existing literature) on these differences, and specifically what conclusions can you draw from differential co-expression of studied hypo-markers with those epi markers. OCT4 is used as an epi marker, but it has been shown to be necessary for differentiation of all three lineages in human embryos.

We thank the reviewer for giving us the opportunity to clarify our choice of epi markers. As mentioned in the reviewer’s comment, OCT4 is expressed in all three lineages and can be observed in the ICM and epi throughout blastocyst development, so we chose this marker for Fig. 1. However, OCT4 is also present in TE until D6 and more importantly for this manuscript, in the hypo until late D6, becoming exclusive to the epi only at D7 (Roode et al., 2012; Niakan and Eggan, 2013; Blakeley et al., 2015). We therefore decided to use SOX2 and NANOG as epi markers in Fig. 3. They exhibit similar expression patterns in epi, as described in Yang et al., 2013. Blakeley et al., 2015 confirmed them as epi markers with SOX2 being upregulated from the 4-cell stage and NANOG from the 4-8 cell stage. Stirparo et al., 2018, defined their RNAseq profile: medium expression in the intermediate population, high expression in epi and low- to no expression in hypo. Comparison between NANOG, OCT4 and hypo markers SOX17 and GATA4 at D7 is presented in Fig. 1D of Roode et al., 2012 and Fig. 6 of Blakeley et al., 2015. In both publications, OCT4 is found in cells also expressing SOX17 at the later stages, but in Roode et al., NANOG is already exclusive to the epi.

© 2023. Published by The Company of Biologists under the terms of the Creative Commons Attribution License (https://creativecommons.org/licenses/by/4.0/).

Therefore, since both NANOG and SOX2 are restricted to the epi and downregulated from hypo precursors earlier than OCT4, we chose these markers to distinguish the point at which the lineages are specified (Roode et al., 2012; Blakeley et al., 2015). We have added this information to the revised manuscript. In our data, we did not observe GATA4+NANOG+/SOX2+ cells after late D6. Interestingly, as mentioned in the manuscript (line 187 in previous version) GATA4+ cells were always found next to the blastocoel, suggesting a physical mechanism correlating with GATA4 expression perhaps in conjunction with biochemical signalling. We were unable to find reference to inner cells expressing GATA4 in the literature (Guo et al., 2021; Meistermann et al., 2021; Roode et al., 2012).

We did not observe high FOXA2, high SOX2/NANOG cells in any of our immunostained embryos, suggesting that once expression of late hypo markers such as FOXA2 is initiated, NANOG and SOX2 expression is rapidly lost, in contrast to OCT4, as was shown in Blakely et al., 2015, Fig. S7. In our initial submission, we detected one NANOG+ FOXA2- GATA4+ cell in 1 of 5 embryos. We have now repeated this antibody combination in additional embryos and found high levels of FOXA2 in some cells expressing very low levels of NANOG. This prompted us to investigate further the co-expression between SOX2 or NANOG and FOXA2 or GATA4. In the violin plots now presented in Figure 3I, we show that at late D6, a percentage of ICM cells downregulates NANOG/SOX2 and upregulates FOXA2 leading to the appearance of hypo precursors. We therefore suggest that FOXA2 expression heralds hypo precursor specification, coinciding with downregulation of NANOG and SOX2. We have added this information to the manuscript.

-Conclusions: l. 223 > Analysis of marker expression may be correlated with blastocyst morphology to help identify the best embryos for transfer to patients < . As much as marker expression analysis provides valuable information about mechanisms of embryonic development, currently it can't be used as a tool for assessing embryo quality in clinical applications.

We do realise that embryos cannot be stained in the clinic prior to transfer. However, ultimately, we hope we may be able to associate subtle morphological features detectable using bright field at early-mid blastocyst stages (when the embryos would normally be chosen for transfer to patients) with specific patterns of marker expression acquired by later stages that predict ‘normal’ development. Thus, by correlating bright field images at early stages with lineage segregation revealed by marker expression of the resulting late blastocysts, we may be able to create a chart to be shared with clinics. However, we take the reviewer’s point that this correlation may not be possible. We have therefore removed this claim from our manuscript and instead inserted the following sentence: ‘Establishing the order of appearance of hypo markers in the context of various levels of epi markers during human blastocyst maturation provides an important benchmark for assessing the utility of blastoids constructed from stem cell lines as a model to study the early stages of human embryo implantation in vitro, since currently these structures tend to exhibit low hypoblast contribution (Kagawa et al., 2022; Yanagida et al., 2021)’.

Reference to other data
-L.114 >as in the mouse embryo< - what stage of mouse embryonic development are you referring to, and which publication

We apologise for not making this clear. E3.5 in mouse would be approximately equivalent to early D6 in human. We refer to Artus et al., 2011 and Plusa et al., 2008. However, in light of the reviewer’s insightful comment, we have removed comparison to the mouse embryo in the text and Fig. 1 because of the relative delay of SOX17 expression at D5 in human embryos.

-L.55 – yolk sac required to pattern the epi – did you perhaps mean hypo required… ? If so, please provide additional references. In addition to TE and hypo, short info on epi function should be also provided

We apologise if this sentence was misleading and have rewritten it to clarify that hypo patterns the epi, but is also the source of yolk sac. We also added the sentence “Epi is the precursor of the embryo proper”. We have supplemented the reference for that statement with an additional publication using human pluripotent stem cells differentiated into human yolk sack-like cells: Mackinlay et al., 2021. While hypo (visceral endoderm) patterning of epi is well documented in mice, only one publication exists to date addressing this question in human embryos. In Mole et al.,

© 2023. Published by The Company of Biologists under the terms of the Creative Commons Attribution License (https://creativecommons.org/licenses/by/4.0/).

2021, the conclusion was based on the existence of CER1+ cells in a subset of hypo at day 9 (post-implantation) which correlated with adjacent epi cells negative for pSMAD1.5.

Other suggestions
-The publication would benefit from discussing these findings with available data on hypoblast formation in other mammals, in addition to mouse, to potentially highlight interspecific similarities.

We appreciate the importance of this point. However, we are already in danger of exceeding the word limit for this short report and will reserve this discussion for a future review article.

-The organization of presented data seems confusing (to me). Please maybe consider starting your results presentation from gene expression analysis?

We thank the reviewer for making this suggestion. However, our rationale for starting with SOX17/OCT4 expression pattern in the embryo was to address the uncertainty regarding the order of lineage segregation in human embryos and highlight that expression of SOX17 could be misleading. Since the existence of an undefined ICM from which both epi and hypo arise was presented in Radley et al., 2022, we sought markers of hypo expressed at earlier stages in the ICM. By exploiting entropy sorting analysis, we found PDGFRA and subsequently confirmed its expression in all ICM cells at D5.

-L.261 refers to unpublished methods – if still unpublished, please provide more detailed information in this manuscript

Fortunately, this paper has now been published and we have updated the reference in the text.

Reviewer 3 Advance Summary and Potential Significance to Field:
Mechanisms that regulate the segregation of the human hypoblast have not been elucidated, despite their fundamental importance for understanding human development and to inform stem cell derived models of early embryonic or extraembryonic cell types. There is widespread interest in the area, especially with the development of advanced integrated stem cell models of human embryos that are notably deficient in hypoblast specification.

There are suggestions that the epiblast, hypoblast and trophectoderm all arise simultaneously (e.g. Petropolous et al., 2016) at the blastocyst stage. Alternatively, it has been proposed that the hypoblast segregates from either the epiblast (Meistermann et al., 2021) or an unspecified inner cell mass (similar to the mouse). In this manuscript the authors perform immunofluorescence analysis of human embryos to investigate the timing of the initiation and segregation of molecular markers of the epiblast and hypoblast.

We thank the reviewer for the positive and constructive summary of our findings and for highlighting the importance of studying hypo segregation in the human embryo.

The take home messages are: 1) the epiblast and hypoblast arise from common ICM progenitor 2) the molecular hierarchy of hypoblast transcription factor expression is PDGFRA expression, followed by SOX17, FOXA2 and GATA4.

Point 1) is important because there has been some confusion that arose from the Meistermann scRNA-seq analysis (and other scRNA seq papers) where the ICM is poorly defined transcriptionally. However, the authors cover some of this in a recent informative paper in a Stem Cell Reports by re-analysing the Meistermann single cell data: https://www.sciencedirect.com/science/article/pii/S2213671122004568?via%3Dihub. In this manuscript the analysis has been expanded to focus on the hypoblast. We think it's interesting to note that while there is just one panel of staining in the Stem Cell Reports paper, the n number of each stage is considerably higher than in this manuscript.

We thank the reviewer for this question about n numbers, which was also brought up by Reviewer 2, encouraging us to clarify this information. In the first submission, we decided to use the n number corresponding to embryos thawed, stained and imaged in the same session, since those

© 2023. Published by The Company of Biologists under the terms of the Creative Commons Attribution License (https://creativecommons.org/licenses/by/4.0/).

were quantified together in the scatter and violin plots. However, we have now realised that these low numbers do not show how many embryos we actually imaged in total for this project. We have corrected this by adding the total number of embryos stained for each antibody combination in the image panel while keeping the number of embryos quantified in the chosen dataset in the scatter plot next to the images. Please see our response to Reviewer 2 for a more detailed explanation.

Also, in response to queries about sufficiency of n numbers, we investigated additional embryos to comlement our results for NANOG expression in comparison to the hypoblast markers. In this new dataset, we observed additional D6 embryos with cells positive for PDGFRA and FOXA2, whilst still negative for GATA4, confirming its later appearance in the sequence of hypoblast activation. Moreover, based on our initial results, we had hypothesized that despite its earlier appearance, FOXA2 expression was not compatible with NANOG. For the additional dataset, we obtained 1 x D5, 2 x early D6, 2 x late D6 and 3 x D7 and observed weak FOXA2 expression in one NANOG-high cell in one embryo. We therefore have decided to remove our statement about FOXA2 expression not being possible in NANOG+ cells. We thank the reviewer for encouraging us to increase our n numbers leading to more robust data.

Point 2) is reminiscent of a publication from Plusa et al 2008: https://doi.org/10.1242/dev.021519 which was also published in Development. This has been an impactful and highly cited study in the field. The biggest difference is that Plusa et al paper contained live-imaging of reporters for Pdgfra and was therefore able to correlate timing, gene expression and morphological formation of the hypoblast layer (but notably, the Plusa et al paper didn't have scRNA-seq analysis, because it hadn't yet been developed).

We were of course inspired by the magnificent Plusa paper and also by Artus et al., 2011 in which the order of appearance for PrE markers in the mouse embryo was determined. This order was then confirmed in Nowotschin et al., 2019 via scRNAseq. Our HFEA license for human embryo work allows us to investigate lineage segregation and stem cell derivation using embryos donated by assisted conception patients with informed consent, but does not include genetic modifications within embryos. Also, most embryos left over from IVF treatment are frozen at D5/D6, so we rarely have access to zygotes. However, we are considering generating a PDGFRA reporter naïve pluripotent stem cell line for use in blastoids in future, which we would make available to the community. Another possibility would be to obtain a fluorescently-conjugated PDGFRA antibody able to label live embryos. We previously used SUSD2 antibody as a live reporter for epiblast (see Bredenkamp et al. 2019; Guo et al., 2021). Unfortunately, under our licence, whole live human embryos need to be locked under restricted access, so cannot be imaged by timelapse in our facility. However, we thank the reviewer for this suggestion. In our manuscript, we combined scRNAseq data and protein visualization via immunostaining at different stages of blastocyst development. We imaged 13 D5 human embryos stained for PDGFRA, 13 early D6, 15 late D6 and 11 D7. We believe these are sufficient numbers to represent blastocyst development.

Reviewer 3 Comments for the Author:
Major points:

We felt that the approach on this manuscript is sound, but with small n numbers, and human embryos being variable, we were not sure whether conclusions could be drawn with certainty with regards to timing of staining (and epiblast/hypoblast ratios). The authors noted that "No statistical analysis was performed due to the low number of embryos per stage per batch" (line 266) - and we therefore wondered if the authors had managed to obtain more embryos for staining that would allow them to perform statistical analysis. If so, this would strengthen the manuscript and conclusions. Also, the novelty in staining of markers and timing/comparison of OCT4, SOX2, NANOG, GATA4, SOX17, OTX2, FOXA2 have been described before.

As outlined above, we hope that the updated total n number will increase the strength of our manuscript, since previously, we had only presented the numbers from one of our datasets. Although not all embryos stained were quantified in detail, they were all scored for lineage contribution to reach our conclusions. Although, as the reviewer points out, SOX17 and OCT4 had been described together before, we have quantified their co-expression at the protein level in individual cells for the first time. In the list of markers mentioned by the reviewer, PDGFRA is missing, which we have discovered is the earliest marker in the context of hypo specification in the

© 2023. Published by The Company of Biologists under the terms of the Creative Commons Attribution License (https://creativecommons.org/licenses/by/4.0/).

human embryo. In addition, FOXA2 and NANOG had not been studied in parallel. We did not detect co-expression between these two markers in our first submission and in a repeat experiment during revision, we noticed that only weak FOXA2 expression can be found in NANOG+ cells. We agree with the reviewer that drawing conclusions from the epi/hypo ratios is complicated by human embryo variability, but we did not use obviously abnormal embryos and therefore consider our data, particularly with the additional embryos immunostained in response to the reviewers' comments, of sufficient value to be worthy of discussion. We consider that the combination of scRNAseq analysis and immunostaining provides an important advance regarding the order of marker expression. Because of the variability inherent in embryos left over from fertility treatment from different clinics, we are dubious that statistical analysis of marker levels will contribute more information at present. However, we believe that visualizing the number of cells co-expressing epi and hypo markers in the scatter plots is novel and informative.

We think it would be important for the finding from this manuscript to be out there, and that there is nothing fundamentally wrong with the paper. The only limitation is that it is descriptive. That said it provides protein expression data to address an important and controversial topic in the field and will therefore be a welcome addition to inform our understanding of the mechanisms underlying the second cell fate specification in human development.

We agree that our manuscript is descriptive, but this is an essential starting point for future work. Our aim is to describe the sequence in which lineage markers are expressed in the embryo and provide comparative quantification, information that will be important for future stem cell-based models for early human development.

Minor points:
1.Please provide details of the objectives and numerical aperture in the methods section.

We thank the reviewer for pointing out this missing detail. All the imaging was performed using a Leica Stellaris microscope. However, confocal images in Fig. 1 and Fig. S2 were performed using a 40x/1.1 water objective, while images in Fig. 3 were taken with a 40x/1.3 oil objective. This difference was unavoidable, since the work spanned our relocation from Wellcome-MRC Stem Cell Institute (Cambridge) to MRC Human Genetics Unit (Edinburgh).

Figure	Objective	Numerical Aperture	Objective Name
1, S2	40x water	1.1	HC PL APO CS2 40x/1.10 WATER
3	40x oil	1.3	HC PL APO CS2 40x/1.30 OIL

2.The methods for software training for embryo images could not be evaluated because the publication Kraunsoe et al. is not yet accessible. In the future it would be helpful to have a few lines describing this further.

The publication Kraunsoe et al., is now available in BiologyOpen (see updated reference list).

3.Consistency in labelling is needed (for example Epi, epi, Hypo, hypo are used at different points throughout the manuscript and methods section)

We thank the reviewer for spotting this oversight and encouraging us to double-check. We found three Epi/Hypo which have been changed into epi/hypo

4.Line 230 - 232: fix the text here where the boxes are errors.

We believe the boxes the reviewer is referring to would be the temperature and the gas percentages, however, we don't see these errors in our document. We have copied the paragraph below and will make sure the final copy does not include errors.
"Supernumerary frozen blastocysts (D5 and D6) were thawed and cultured in N2B27 medium (Table 1) under mineral oil in a humidified incubator at 37oC, 7% CO_2 and 5% O_2 until reaching the desired stage of development from E5 to E7. Embryonic stage was assessed based on thinning of the zona pellucida and blastocoele expansion (Fig. S1). "

© 2023. Published by The Company of Biologists under the terms of the Creative Commons Attribution License (https://creativecommons.org/licenses/by/4.0/).

5.Detail are needed of how the in house N2B27 is made. This is the basis of the human embryo culture media used.

We appreciate the reviewer comment since different groups used different N2B27 formulae, some of which include serum. As clarification, we have added a table with the recipe to prepare N2B27 media, based on Mulas et al., 2019 https://journals.biologists.com/dev/article/146/6/dev173146/49077/Defined-conditions-for-propagation-and

Reagent	Provider – Cat no	Final concentration
DMEM/F-12	Thermo Fisher – 21331-020	
Neurobasal	Thermo Fisher - 21103049	
B27 (50X)	Invitrogen - 17504044	0.5X
N2	Made in house (ref)	1X
ß-mercaptoethanol (50mM stock)	Thermo Fisher - 31350-010	50µM
L-glutamine (200mM stock)	Thermo Fisher - 25030081	2mM

We have left N2 as "made in house" in the manuscript because both the Sigma commercial version - Cat no: 17502001- and our "in house" version (made at the Wellcome-MRC Stem Cell Institute) have the same recipe, including preparation instructions which can be consulted in Mulas et al., 2019.

However, we have added the table with each component to our response to the reviewer and will be happy to add it to the final manuscript if requested.

Reagent	Provider - Cat no	Final concentration
Apotransferrin (100mg/ml)	Sigma -Aldrich - T1147<br>Bio -Techne - 3188 -AT	10mg/ml
DMEM-F12	Sigma-Aldrich - D6421	1X
Insulin (10mg/ml)	Various	2.5mg/ml
BSA fraction (7.5%)		0.75%
Sodium selenite (3Mm)	Sigma-Aldrich - S5261- 10G	3µM
Putrescine (160mg/ml)	Sigma-Aldrich - P5780-5G	1.6mg/ml
Progesterone (0.6mg/ml)	Sigma-Aldrich - P8783-1G	1.98µg/ml

6.Line 58: cite the HFEA reference metrics used in this sentence.

We obtained the information from the HFEA website, the link is copied below. The information is frequently updated and it is published in their website to facilitate access to patients.

"As figure 1 shows, birth rates from IVF have steadily increased over time with the average birth rate per embryo transferred standing at 24% in 2018, compared with just 7% in 1991."

https://www.hfea.gov.uk/about-us/publications/research-and-data/fertility-treatment-2019-trends-and-figures/

Note: Figure provided for reviewers has been removed. It showed Figure 1 from Human Fertilisation & Embryology Authority (May 2021). Fertility treatment 2019: trends and figures: UK statistics for IVF and DI treatment, storage, and donation [online]. Available from: https://www.hfea.gov.uk/about-us/publications/research-and-data/fertility-treatment-2019-trends-and-figures/

© 2023. Published by The Company of Biologists under the terms of the Creative Commons Attribution License (https://creativecommons.org/licenses/by/4.0/).

7.Line 70: Remove the reference Zhu and Zernicka-Goetz, 2020 in this sentence – this is a review on the regulative mechanisms of mammalian development and speaks to Carm1 paraspeckles; similarly we don't believe the Meistermann does not investigate IF markers that distinguish TE versus ICM, nor do they investigate polarity acquisition or Hippo signalling therefore this reference should also be removed here.

We thank the reviewer for noticing these two mistakes. We have changed the reference of Zhu and Zernicka-Goetz, 2020 to Zhu et al., 2021 which is the reference we were intending to use, and have removed Meistermann et al., from the sentence.

8.Lines 79-82: reference Blakeley et al 2015 (see Fig. 5) is needed where the enrichment of transcripts in the PrE/hpoblast such as GATA4, GATA6, SOX17, PDGFRA, COL4A1, SPARC and FOXA2 were noted.

We thank the reviewer for pointing out this detail. We have included this reference in the manuscript for both the IF and scRNAseq statements.

Second decision letter

MS ID#: DEVELOP/2022/201522

MS TITLE: Evidence implicating sequential commitment of the founder lineages in the human blastocyst by order of hypoblast gene activation

AUTHORS: Elena Corujo-Simon, Arthur H Radley, and Jennifer Nichols
ARTICLE TYPE: Research Report

I am happy to tell you that your manuscript has been accepted for publication in Development, pending our standard ethics checks.

Reviewer 1

Advance summary and potential significance to field

This is an interesting and well-executed work, in which the authors use previously generated single-cell RNA-seq data from human preimplantation embryos, to identify genetic components and markers involved in the primitive endoderm/hypoblast lineage.

Comments for the author

The authors have provided a compelling response to the criticisms previously raised by this reviewer, and explained in detail the reasons for some of the limitations of their study as are the sparsity of biological material, and the lack of antibodies know to work on human embryos. It is clear that without enough embryos, it is difficult to test new reagents. The authors have also addressed in detail the concerns and suggestions of the other reviewers.

Although some of the concerns regarding the confirmatory nature of much of the results are still true, this reviewers understands the contribution of this report to the study of human preimplantation development, and therefore its relevance.

Reviewer 2

Advance summary and potential significance to field

© 2023. Published by The Company of Biologists under the terms of the Creative Commons Attribution License (https://creativecommons.org/licenses/by/4.0/).

This study provides new information on the sequence of genes activated during human hypoblast specification and identifies PDGFRA as the earliest hypoblast specific marker in humans.
This manuscript provides mostly descriptive data, yet novel and scientifically sound, so it will be valuable to scientific community, as the availability of any data regarding human embryos is very limited.

Comments for the author

All the previous comments have been satisfactorily addressed by the authors and all the necessary corrections have been introduced.

One minor point: Figure 1A - there is n= missing in the D7 panel.

Reviewer 3

Advance summary and potential significance to field

The authors have addressed all of our comments.

Comments for the author

The manuscript and data are much improved and more robustly support the conclusions.

© 2023. Published by The Company of Biologists under the terms of the Creative Commons Attribution License (https://creativecommons.org/licenses/by/4.0/).
